# Deep sequencing of small RNAs specifically associated with Arabidopsis AGO1 and AGO4 uncovers new AGO functions

**DOI:** 10.1111/j.1365-313X.2011.04594.x

**Published:** 2011-05-10

**Authors:** Huan Wang, Xiuren Zhang, Jun Liu, Takatoshi Kiba, Jongchan Woo, Tolulope Ojo, Markus Hafner, Thomas Tuschl, Nam-Hai Chua, Xiu-Jie Wang

**Affiliations:** 1 Laboratory of Plant Molecular Biology, The Rockefeller UniversityNew York, NY 10065, USA; 2 State Key Laboratory of Plant Genomics, Institute of Genetics and Developmental Biology, Chinese Academy of SciencesBeijing, 100101, China; 3 Graduate University of the Chinese Academy of SciencesBeijing, 100101, China; 4 Department of Biochemistry and Biophysics, Institute for Plant Genomics and Biotechnology, Norman Borlaug Center 112A, 2123 TAMU, Texas A&M UniversityCollege Station, TX 77843, USA; 5 RIKEN Plant Science CenterSuehiro 1-7-22, Tsurumi, Yokohama 230-0045, Japan; 6 Howard Hughes Medical Institute, Laboratory of RNA Molecular Biology, The Rockefeller University1230 York Avenue, New York, NY 10065, USA

**Keywords:** AGO1, AGO4, microRNA, *trans*-acting siRNAs, nat-siRNA, post-transcriptional gene silencing

## Abstract

As important components of small RNA (smRNA) pathways, Argonaute (AGO) proteins mediate the interaction of incorporated smRNAs with their targets. Arabidopsis contains 10 AGO proteins with specialized or redundant functions. Among them, AGO1 mainly acts in microRNA (miRNA) and small-interfering RNA (siRNA) pathways for post-transcriptional gene silencing (PTGS), whereas AGO4 regulates transcriptional gene silencing (TGS) via endogenous 24-nucleotide (nt) smRNAs. To fully characterize smRNAs associated with AGO1 and AGO4, we developed a two-step protocol to purify AGO/smRNA complexes from flowers, leaves, roots and seedlings with enhanced purity, and sequenced the smRNAs by Illumina technology. Besides recovering most previously annotated smRNAs, we also identified some additional miRNAs, phased smRNA clusters and small-interfering RNAs derived from the overlapping region of natural antisense transcript pairs (NAT) (nat-siRNAs). We also identified a smRNA distribution feature on miRNA precursors which may help to identify authentic miRNAs. Organ-specific sequencing provided digital expression profiles of all obtained smRNAs, especially miRNAs. The presence and conservation of collateral miRNAs on known miRNA precursors were also investigated. Intriguingly, about 30% of AGO1-associated smRNAs were 24-nt long and unrelated to the 21-nt species. Further analysis showed that DNA-dependent RNA polymerase IV (Pol IV)-dependent smRNAs were mainly 24 nt and associated with AGO4, whereas the majority of the potential Pol V-dependent ones were 21-nt smRNAs and bound to AGO1, suggesting the potential involvement of AGO1 in Pol V-related pathways.

## Introduction

Small non-coding RNAs (smRNAs) have recently emerged as pivotal regulators in plant growth and development, adaptation to the environment and tolerance to biotic stresses. Two major classes of smRNAs in plants are micro-RNAs (miRNAs) and small-interfering RNAs (siRNAs). Mi-RNAs are processed by a Dicer-like enzyme from imperfectly self-folded hairpin precursors transcribed from *miRNA* genes (Kurihara *et al.*, 2006; Song *et al.*, 2007). Small-interfering RNAs are processed from double-strand RNA duplexes or long RNA transcripts with inverted complementarity, and numerous endogenous siRNAs have been found in plants (Meister and Tuschl, 2004). According to their origins, some plant endogenous siRNAs could be further grouped into different classes: repeat-associated siRNAs (ra-siRNAs) generated from transposons, heterochromatic and repetitive genomic regions; nat-siRNAs derived from the overlapping regions of natural antisense transcript pairs (NAT); and *trans*-acting siRNAs (ta-siRNAs) initiated by miRNA cleavage of target mRNAs (Allen *et al.*, 2005). Besides, there are also large numbers of smRNAs without clear structural features and functions (Chellappan *et al.*, 2010; Chen *et al.*, 2010).

Mature smRNAs are incorporated into a ribonucleoprotein complex termed the RNA-induced silencing complex (RISC) to regulate the expression of target genes at transcriptional and post-transcriptional levels in a sequence-specific manner (Bartel, 2004; Vaucheret, 2008). The central component of RISCs is AGO protein, which recruits miRNAs/siRNAs to interact with target mRNA or DNA sequences to execute their functions.

*Arabidopsis thaliana* possesses 10 AGO family proteins classified into three phylogenic clades. AGO1, AGO5 and AGO10 belong to the first clade; AGO2, AGO3 and AGO7 form the second clade; and the rest are the third group (Vaucheret, 2008). Genetic studies have shown that AGO1 is indispensable for miRNA pathways, as *ago1* null mutants showed decreased number and abundance of detectable miRNAs and increased expression of corresponding target mRNAs (Vaucheret *et al.*, 2004). Several classes of siRNAs, including transgene siRNAs, virus siRNAs and ta-siRNAs, also associate with AGO1 complexes (Baumberger and Baulcombe, 2005; Qi and Hannon, 2005; Zhang *et al.*, 2006b; Montgomery *et al.*, 2008b). It has been shown that the 5′ terminal nucleotide of a smRNA directs its AGO destination, and AGO1-associated smRNAs are mainly 21-nt long with the 5′-first nucleotide biased towards uridine (Mi *et al.*, 2008; Takeda *et al.*, 2008).

AGO4 regulates epigenetically silent states of repeated loci, transposons and heterochromatin regions through its associated 24-nucleotide (nt) siRNAs (Vaucheret, 2008). DNA-dependent RNA polymerase IV (Pol IV) and V (Pol V) as well as several other proteins also participate in this process (Matzke *et al.*, 2009). Small non-coding RNAs immunopurified with AGO4 are predominantly 24-nt long with a strong preference for 5′ terminal nucleotide of adenine (Mi *et al.*, 2008).

Although genome-wide profiling of AGO1- and AGO4-associated smRNAs have been reported previously, the studies were based on an early stage of parallel pyrosequencing technology with low sequencing depth (Qi *et al.*, 2006; Mi *et al.*, 2008). In addition, the tissue-specific expression profiles of miRNAs and other smRNAs were not well investigated. Here, we used extracts from Arabidopsis flowers, leaves and roots as well as 10-day-old seedlings to purify smRNAs associated with AGO1 and AGO4 protein complexes using a two-step immunoprecipitation method. Sequencing results of these smRNAs revealed unexpectedly that about 30% of the AGO1-associated smRNA species were 24-nt in length. Genome mapping studies suggested potential functions of these AGO1-bound 24-nt smRNAs in mediating transcriptional gene silencing (TGS). The work also provided a detailed tissue-specific expression profile of miRNAs. Besides known functional smRNAs, we also identified additional miRNAs, collateral miRNAs encoded in known miRNA precursors, nat-siRNAs and phased siRNA clusters.

## Results

### Isolation of AGO1/4-containing ribonucleoprotein complexes

**Figure 1:**
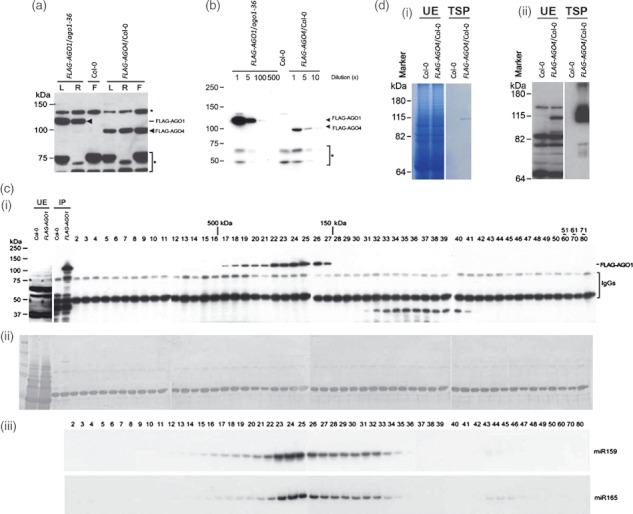
Isolation of AGO1/4–small non-coding RNA (smRNA) complexes. (a) Expression patterns of FLAG-AGO1 and FLAG-AGO4. Total protein extracts (30 μg) were analyzed by western blot using FLAG antibody. L, leaves; R, roots, F, flowers. Non-specific cross-reacting bands are indicated by (*). Arrow heads indicate specific FLAG-AGO bands. (b) Relative abundance of AGO1 and AGO4 proteins in flowers. A series of dilution were made for AGO1 and AGO4. Non-specific cross-reacting bands (*) serve as internal controls. (c) Fractionation of crude extracts containing FLAG-AGO1 by gel filtration. (i) The first two lanes from the left show western blot analysis of unfractionated extracts (UE) of Col-0 control and *FLAG-AGO1/ago1-36.* The next two lanes show single-step immunoprecipitation of the two samples (Col-0 and *FLAG-AGO1/ago1-36*) using FLAG antibody. Note that FLAG-AGO1 has a molecular mass of approximately 120 kDa. The unfractionated extracts from *FLAG-AGO1/ago1-36* were fractionated by gel filtration and 80 fractions (numbers shown) were collected. An aliquot of each fraction was immunoprecipitated with M2-agorase antibody and purified FLAG-AGO1 was detected by western blot using a different FLAG antibody. (ii) The same membrane was stained with Coomassie brilliant blue to monitor the size of FLAG-AGO1. (iii) Another aliquots of gel filtration fractions were used for smRNA blot analyses of miR159 and miR165. (d) Two-step purification of AGO4-smRNA complexes. (i) Unfractionated extracts (UE) and samples derived from two-step purification (TSP) were analyzed by SDS gel stained with Coomassie brilliant blue. (ii) Western blot analysis of unfractionated extracts (UE) and samples derived from TSP using an anti-FLAG antibody.

Transgenic plants expressing *P*_*AGO1*_*-FLAG-AGO1/ago1-36* (Baumberger and Baulcombe, 2005) and *P*_*AGO4*_*-FLAG-AGO4/Col-0* genes were used. Western blot analysis showed that the FLAG-AGO1 expression level was about 1.4 to 2.7 times higher than that of FLAG-AGO4 in leaves and roots, and about 13.3 times higher than in flowers (Figure 1a,b and Table S1 in Supporting Information).

Immunoprecipitation (IP) has been widely used to isolate AGO protein/smRNA complexes (Mi *et al.*, 2008; Montgomery *et al.*, 2008a). However, owing to the high homology of AGO family proteins and low specificity of antibodies, complexes isolated by single-step IP may still contain contaminating proteins caused by non-specific cross-reactions (Figure 1c, IP lanes). To obtain AGO complexes of high purity, we developed a two-step purification (TSP) protocol. Protein extracts from *P*_*AGO1*_*-FLAG-AGO1/ago1-36* seedlings were first fractionated through a gel filtration column and fractions 17–27 containing FLAG-AGO1 (Figure 1c) were used for further purification by IP and smRNA isolation. Since AGO1 is the dominant player in the miRNA pathway, we hypothesized that miRNAs should be co-fractionated with it. Figure 1(c) shows that miR159 and miR165 were distributed in a broader range of fractions than the FLAG-AGO1, indicating the association of these miRNAs with other AGO complexes. Using the TSP protocol we also obtained FLAG-AGO4 complexes of high purity (Figure 1d).

### Identification and characterization of smRNAs associated with AGO1/AGO4-smRNA complexes

Small non-coding RNAs of purified AGO1/AGO4 complexes from seedlings and three different organs were subjected to deep sequencing (Figure S1). Unfractionated smRNAs from the corresponding samples of non-transformed wild-type (WT) plants were also sequenced. Each sample yielded approximately 3–6 million total reads of smRNA sequences. After removal of adaptor sequences, reads with lengths between 19- and 28-nt (2.5–5.4 million per sample) were further processed. Approximately 57–89% of the reads were mapped perfectly to the Arabidopsis genome and included in our analysis (Table S2).

To examine whether the TSP method indeed produced better results, we also obtained AGO1-associated smRNA sequences from Arabidopsis flowers and roots by the IP method. Three to 5 million smRNAs were obtained from both the IP and TSP samples. Nearly all smRNAs in the IP samples with clone numbers no less than 10 were included in the TSP samples, whereas only 60–70% of smRNAs with the same clone number threshold in the TSP samples were detected in the IP samples (Figure S2A,B). Therefore, the TSP method is more sensitive and robust in identification of smRNAs than IP purification, especially for low-abundance smRNAs. The increased output of Illumina sequencing technology and the separation of AGO-associated smRNAs from different organs allowed us to obtain smRNA populations much larger than those reported previously (Qi *et al.*, 2006; Mi *et al.*, 2008). More than 50% of AGO1-associated smRNAs and 95% of AGO4-associated smRNAs (both with clone numbers no less than 10) reported previously (Mi *et al.*, 2008) were included in our data sets (Figure S2C,D). With the same threshold, only about 17% of AGO1-associated smRNAs and 26% of AGO4-associated smRNAs in our data sets were identified previously (Mi *et al.*, 2008), and the proportion was even lower for smRNAs with fewer clone numbers.

**Figure 2:**
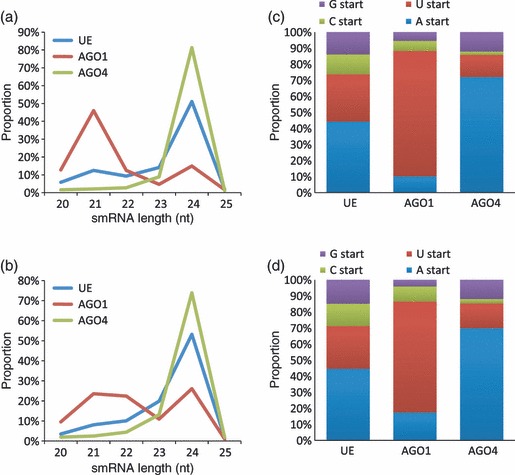
Size distribution and first nucleotide preference of small non-coding RNAs (smRNAs) from flowers in the unfractionated sample and AGO1/AGO4 complexes. The first two panels show the size distribution of total (a) and non-redundant (b) smRNAs. The *x*-axis presents the length of smRNA (in nucleotides, nt) and the *y*-axis presents the proportion of each smRNA class. The last two panels show the relative abundance of total (c) and non-redundant (d) smRNAs with different 5′ terminal nucleotides and their AGO association. UE, unfractionated samples.

As reported previously, total smRNAs extracted from plants without AGO affinity purification were dominated by 21-nt and 24-nt long species, with the population of 24-nt smRNAs much larger than the 21-nt ones. The majority of AGO1-associated smRNAs were 21-nt long (Figures 2 and S3). By contrast, AGO4-associated smRNAs were overwhelmingly dominated by 24-nt species, with a clear depletion of 21-nt smRNAs. Surprisingly, a large group of 24-nt smRNAs were also detected in all AGO1 affinity purified samples (accounting for 10–15% of total AGO1-associated smRNAs and 23–42% of non-redundant AGO1-associated smRNAs; Table S2). Both the 21-nt and 24-nt smRNAs were also detected in the purified AGO1 sample by ethidium bromide staining (Figure S1). The number of 21-nt smRNAs in the AGO1-associated total smRNA population reduced significantly when non-redundant sequences were analyzed, but that of the 24-nt smRNAs did not change much. This result indicates that AGO1-associated 24-nt smRNAs comprised a large number of diversified sequences with low expression abundance (Figures 2a,b and S3). Consistent with previous reports (Mi *et al.*, 2008; Takeda *et al.*, 2008), strong preferences for the 5′-first nucleotide of ‘U’ for AGO1-associated smRNAs and ‘A’ for AGO4-associated smRNAs were observed for both total and non-redundant smRNA sequences (Figures 2c,d and S3).

### Differences between AGO1 and AGO4-associated 24-nt smRNAs

To investigate the functional relationship of AGO1 and AGO4, we compared smRNAs preferentially associated with either AGO. We considered a smRNA as dominantly associated with AGO1 if its normalized clone number in the AGO1 sample was at least five times larger than that in the AGO4 sample, and vice versa. We further separated the Arabidopsis genomic sequences into 100-nt sub-regions and calculated the total AGO dominance of each region individually. The genomic loci of about 95.9% of AGO1-dominant 21-nt smRNAs and 95.7% of AGO4-dominant 24-nt smRNAs did not overlap, with most AGO4-associated 24-nt smRNAs being derived from heterochromatin regions, transposons and pseudogenes.

As the AGO1-associated 24-nt smRNAs were not well studied previously, we first examined their relationships with the 21-nt ones. Only 1.3% of AGO1-dominant 24-nt smRNAs were produced from the same loci as 21-nt smRNAs, but with much lower clone numbers (Table S3). By contrast, 99.6% of AGO4-dominant 21-nt smRNAs overlapped with 24-nt ones (Table S3). These observations suggested that the AGO1-dominant 21-nt and 24-nt smRNAs have different genomic origins, whereas the AGO4-preferred 21-nt smRNAs are mainly length isovariants of the 24-nt ones.

**Figure 3:**
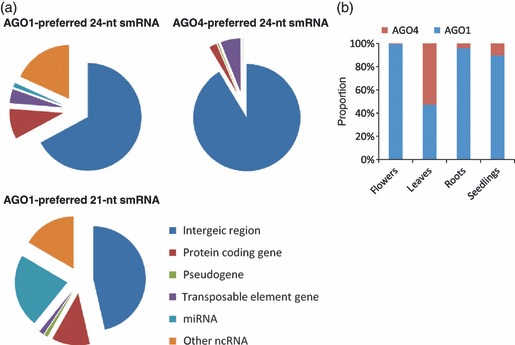
Genomic features of AGO1- and AGO4-preferred smRNA species. (a) Genomic features of loci preferentially generating AGO1/AGO4-preferred 21- and 24-nucleotide (nt) small non-coding RNAs (smRNAs). (b) AGO-preference of 24-nt smRNAs derived from tRNA, rRNA, small nuclear RNA (snRNA) and small nucleolar RNA (snoRNA). The *y*-axis presents the proportion of smRNAs preferentially associated with each AGO.

Moreover, although around 70% of genomic loci giving rise to AGO1-preferred 24-nt smRNAs were located in intergenic, heterochromatin or centromeric regions (Figure 3a), only 5% of AGO1-preferred 24-nt smRNA loci overlapped with those producing AGO4-preferred smRNAs. Therefore, the AGO1-preferred 24-nt smRNAs were unique and not due to contamination by the AGO4-preferred ones. Interestingly, 24-nt smRNAs derived from tRNAs, rRNAs, small nucleolar RNAs (snoRNAs) and small nuclear RNAs (snRNAs) were preferentially identified in the AGO1 but not the AGO4 pull-down samples (Figure 3b). These housekeeping non-coding RNA (ncRNA)-derived 24-nt smRNAs accounted for 2–22% of AGO1-associated 24-nt smRNAs in the examined samples.

### Classification of known miRNAs and identification of new miRNAs

**Figure 4:**
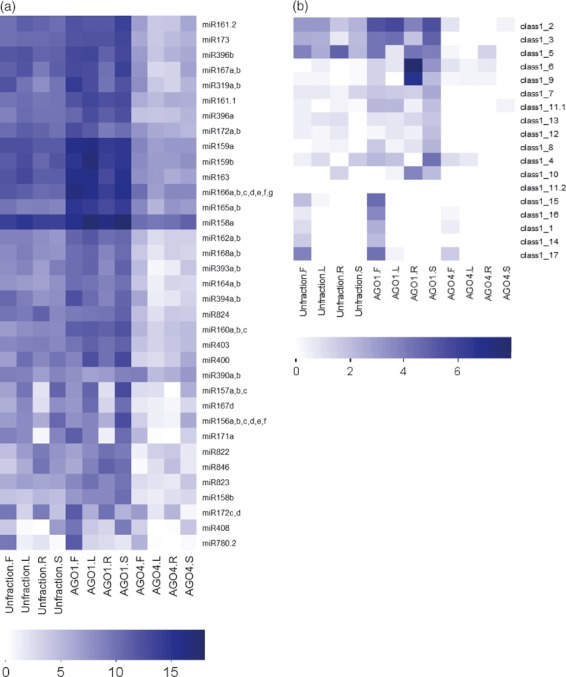
Expression heat map of known and new microRNAs (miRNAs). (a) Expression heat map of known miRNAs with clone numbers no less than 1000 in at least one sample. (b) Expression heat map of new miRNAs. F, flowers; L, leaves; R, roots; S, seedlings. Log_2_ values of normalized clone numbers of each miRNA are presented using color schemes below each panel. The miRNA abundance is positively correlated with the color intensity.

Among the 224 Arabidopsis miRNAs/miR*s recorded in the miRBase (version 15), 198 were detected in one or more samples. Most miRNAs exhibited a strong preference for AGO1 and were depleted in the AGO4 complexes (Figures 4 and S4). The characterization of smRNAs from various tissues enabled us to investigate the organ-specific expression of miRNAs. Overall, the founding members of Arabidopsis miRNAs exhibited much higher expression in all examined samples than those identified later. Among our samples, roots contained the lowest abundance and diversity of miRNAs (Figure 4). Table S4 shows the detailed expression of all detected miRNAs.

**Figure 5:**
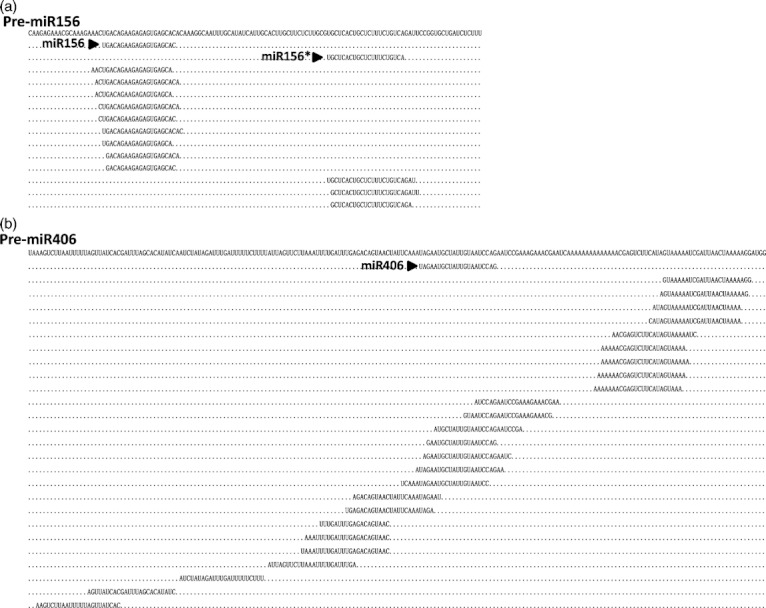
Examples of small non-coding RNA (smRNA) distribution on microRNA (miRNA) precursors. (a) Centered distribution of smRNAs around miR156 and miR156* (indicated by arrow heads) on pre-miR156. (b) Smeared distribution of smRNAs on pre-miR406. Nucleotide sequences outside of the cloned smRNAs on the miRNA precursors are presented by dots.

By mapping all cloned smRNAs to the precursors of annotated miRNAs, we observed that for most known miRNA precursors, perfectly matched smRNAs were centered around the mature miRNA sequences, and in certain cases, miRNA* sequences (Figure 5a, Table S5). By contrast, for precursors of 25 previously annotated miRNAs, cloned smRNAs covered almost every nucleotide of the precursors (Figure 5b, Table S5). Furthermore, these annotated miRNAs usually had low clone numbers and showed an equal or stronger preference for AGO4 rather than AGO1. Therefore, we proposed that these miRNAs may not be *bona fide* miRNAs, but rather siRNAs, although their ‘precursors’ also exhibited hairpin shaped secondary structures. Ten annotated miRNA precursors had no detectable smRNAs in any of the examined samples (Table S5).

The above results led us to establish the following criteria for searching for new miRNAs, which were modified from previously published rules (Meyers *et al.*, 2008): (i) cloned smRNAs should be derived from hairpin-shaped precursors; (ii) cloned smRNAs should exhibit a clear preference for AGO1; (iii) the majority of all cloned smRNAs from the hairpin-shaped precursors should center around the putative miRNA and/or miRNA* sequences; (iv) miR* sequences should also be detected. According to these criteria, 18 candidates with clone numbers no less than 10 in one or more samples were identified and referred as new miRNAs (Figure 4b and Table S6).

### Collateral miRNAs and miRNA variants

**Figure 6:**
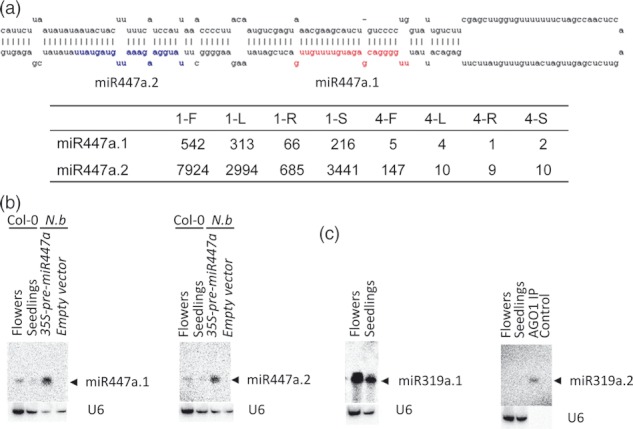
Two microRNAs (miRNAs) generated by the same miRNA precursor. (a) The sequence of miR447a.1 is highlighted in red and that of miR447a.2 in blue. ‘1’ and ‘4’ represent AGO1 and AGO4, respectively. F, flowers; L, leaves; R, roots and S, seedlings. Raw clone numbers in each sample are given. (b) Detection of two miRNAs derived from the precursor of miR447a by smRNA northern blot hybridization. Both miR447a.1 and miR447a.2 can be detected in Arabidopsis flowers and seedlings (10 μg total RNA) as well as *Nicotiana benthamiana* leaves which were transfected with 35S-pre-miR447a construct (2 μg total RNA). (c) Detection of miR319a.1 and miR319.2 by smRNA blot analysis. Each lane contained 10 μg total RNA. U6 RNA was used as a loading control. AGO1 complex was immunoprecipitated using an anti-FLAG monoclonal antibody from flower extracts (AGO1 IP).

A second miRNA species was reported to be encoded in the precursors of miR159 and miR319 (Talmor-Neiman *et al.*, 2006; Axtell *et al.*, 2007; Arenas-Huertero *et al.*, 2009). Here, we also found second miRNA species with high clone numbers on the precursors of miR319a/b, miR447a/b, miR836 and miR868, as well as one new miRNA, namely class1_11. These collateral miRNAs did not overlap in sequence with the reported miRNAs or miRNA*s; some of them were located next to (e.g. class1_11.2) or 21-nt apart (e.g. miR447.2) from the reported miRNAs (Figure 6a and Table S7). Corresponding miRNA*s were also identified for six collateral miRNA species.

The sequence of miR319b.2 was 2-nt shorter than that of miR319a.2. Phylogenetic analysis revealed that the sequences of both miR319a.2 and miR319b.2 were conserved in other dicots and monocots with available sequences, including wine grape, black cottonwood, soybean and rice. This is consistent with a recent report (Zhang *et al.*, 2010). Both miR319a.1 and miR319a.2 were detected in flowers and seedlings, with the former expressed at a higher level. Although miR319a.2 was barely detected in unfractionated samples, it could be detected in a purified AGO1 sample (Figure 6c). Some collateral miRNA species showed a much higher expression than their corresponding primary miRNAs, as exemplified by miR447.2 whose clone number was about 11 times higher than miR447.1 in the AGO1 complex. The expression of miR447.1 and miR447.2 was confirmed by smRNA northern blot hybridization (Figure 6b).

Sequence variants (with shift, deletion or extension on sequences) with clone numbers no less than 10 were detected for 12 known miRNAs. For 11 of them, higher expression of miRNA variants were observed in at least one examined sample, indicating that the miRNA variants might be the functional forms in those samples (Table S8). Organ-specific expression profiles were also observed for the variants of some miRNAs, most of which were in concert with the expression profiles of their corresponding miRNAs.

### Identification of *trans*-acting siRNAs and phased smRNA clusters

**Figure 7:**
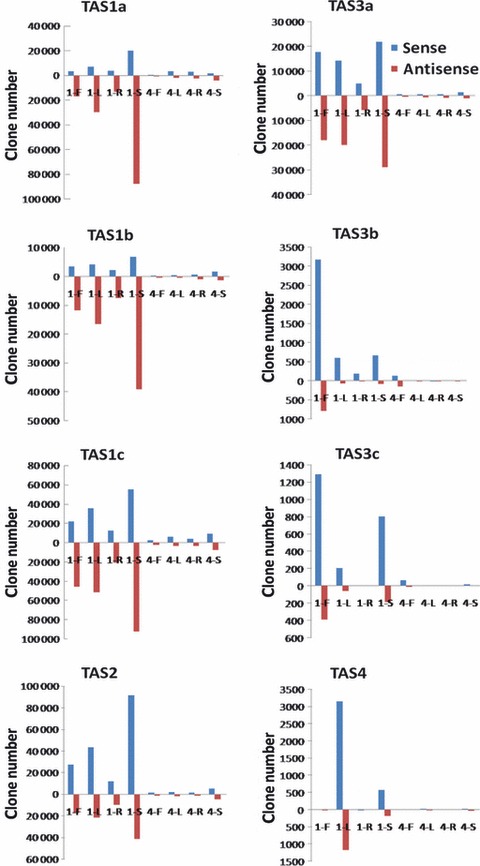
Phased small non-coding RNA (smRNA) clusters on *TAS1-4* genes. Bar charts present total clone numbers of phased smRNAs on the plus strand (blue bars) and minus strand (red bars) of known *TAS* genes in each sample. ‘1’ and ‘4’ represent AGO1 and AGO4, respectively. F, flowers; L, leaves; R, roots; S, seedlings.

Previous reports showed that transcripts of four *TAS* gene families (*TAS1* to *TAS4*) generate ta-siRNAs following cleavage by miRNAs (Montgomery *et al.*, 2008a,b; Felippes and Weigel, 2009). Because ta-siRNAs are processed sequentially from *TAS* transcript derived double-stranded (ds) RNAs, the cloned ta-siRNAs should exhibit in-phase positional patterns. We were able to recover phased ta-siRNAs for all *TAS1-4* family member transcripts from our samples. Overall, the ta-siRNAs derived from *TAS1a-c*, *TAS2* and *TAS3a* were at least 10 times more abundant than those from *TAS3b*, *TAS3c* and *TAS4.* The expression of these ta-siRNAs exhibited strong organ preferences (Figure 7).

Most ta-siRNAs were 21-nt ‘U-start’ smRNAs and predominantly associated with AGO1, with very few ta-siRNAs also identified in the AGO4 complex (Figure 7). The AGO selection of most ta-siRNAs exhibited a strong strand preference. Although similar numbers of non-redundant ta-siRNAs were detected on both the sense and antisense strands of all known TAS genes (Vazquez *et al.*, 2004; Allen *et al.*, 2005; Lu *et al.*, 2006) (Figure S5), the total number of ta-siRNAs identified in our experiments were mainly derived from one strand of *TAS* transcripts (Figure 7).

In addition to known ta-siRNAs, 18 phased smRNA clusters with a unit size of 21-nt were identified from intergenic regions (Table S9). Most phased smRNAs were preferentially associated with AGO1 and enriched in flowers. Small non-coding RNAs from three clusters are more abundant in unfractionated samples, which suggested that they might load in other AGO complexes.

### NAT-siRNAs

Bioinformatic analyses have previously predicted 2660 pairs of potential *cis*- and *trans*-NAT in Arabidopsis (Wang *et al.*, 2005, 2006), which are potential sources for nat-siRNAs. By mapping AGO1/4-associated smRNAs to the previously predicted Arabidopsis *cis*-NAT pairs, we found that 62 *cis*-NAT pairs contained perfectly matched smRNA sequences (Table S10). These smRNAs were mainly derived from the sequence complementarity regions of NAT pairs, and more than half of them exhibited a stronger affinity to AGO1. Organ-specific expression was also observed for some nat-siRNAs.

To examine whether the pairing of NAT transcripts was required for the production of their associated smRNAs, we calculated the smRNA density on different sequence regions. The average density of smRNAs on the overlapping regions of *cis-*NAT pairs was 2.29 times higher than that on all other gene transcripts, with a *P*-value of 2.085 × 10^−11^ as evaluated by the Mann–Whitney *U*-test.

### Possible relationship with Pol IV and Pol V of AGO1/4-dominant smRNAs

Recent studies have shown that Pol IV and Pol V are involved in RNA-directed RNA methylation (RdDM). Pol IV is thought to play a role in smRNA biogenesis, whereas Pol V is reported to produce long non-coding transcripts from intergenic regions (Zhang *et al.*, 2007; Pikaard *et al.*, 2008). To investigate the AGO association of Pol IV/Pol V-dependent smRNAs, we compared our data with the reported loci of Pol IV-dependent and potential Pol V-dependent smRNA biogenesis (Mosher *et al.*, 2008). To ensure accuracy, only smRNAs with raw clone numbers more than five were selected for further analysis, which corresponded to around 28% of the total smRNAs. These smRNAs belonged to three categories: Pol IV (NRPD1a)-dependent, potential Pol V (NRPD1b)-dependent and potential Pol IV and Pol V co-dependent. Consistent with previous reports, smRNAs mapped to the potential Pol IV and Pol V co-dependent loci were mainly 24-nt long and associated with AGO4 (96% were AGO4-preferred), so were Pol IV-dependent smRNAs (92% were AGO4-preferred). We note that 96% of the potential Pol V-dependent smRNAs preferred to associate with AGO1, and majority of which were 21-nt long and with 5′ U (Figures S7 and S8). This observation suggested that AGO1 may also be involved in Pol V-mediated gene silencing pathway via 21-nt smRNAs.

## Discussion

With the continuous development of next-generation sequencing technologies, the number of sequences obtained from each reaction has been increasing significantly. In the pioneering studies of Arabidopsis AGO functions, the 454 and early stage Illumina sequencing technologies have been applied to identify small RNAs associated with AGO1 and AGO4 complexes (Qi *et al.*, 2006; Mi *et al.*, 2008). Here, we implemented a two-step purification strategy to obtain AGO–smRNA complexes of high purity; moreover, we determined smRNA sequences from AGO complexes of seedlings and three different organs. Aided by the improvement of Illumina sequencing technology, we were able to obtain 3–6 million total reads from each sample. Such more in-depth sequencing results and the aggregation of datasets not only recovered most previously reported smRNAs, but also uncovered millions of unknown ones. The combined results enabled us to identify several uncovered features of AGO functions and their associated smRNAs.

### Discovery of miRNAs

Thus far, more than 200 miRNA genes and numerous other types of smRNAs have been identified in Arabidopsis by classic cloning, computational prediction and pyrosequencing approaches (Zilberman *et al.*, 2003; Bartel, 2004; Sunkar and Zhu, 2004; Kasschau *et al.*, 2007; Adachi *et al.*, 2009; Grant-Downton *et al.*, 2009; Hsieh *et al.*, 2009; Chellappan *et al.*, 2010; Fahlgren *et al.*, 2010; Ma *et al.*, 2010). One outstanding issue is whether screening of miRNAs and other classes of smRNAs in Arabidopsis has already reached saturation. Given that thousands of miRNA genes have been identified in mammalian genomes, and this number continues to increase (Landgraf *et al.*, 2007), we believe that additional miRNAs would emerge if specific organs were analyzed or a more powerful sequencing technology applied.

Isolation of AGO/smRNA complexes followed by characterization of the smRNA population is a robust tool for identification of smRNAs. Irrespective of their origins, the smRNAs are eventually channeled into the AGO ‘sink’. The enrichment of smRNAs in presumably functional AGO complexes provides an important advantage for thorough identification of smRNAs, especially the low-abundant ones. Here, we have identified 18 miRNA candidates. Although these miRNA candidates are likely to be functional as they were recovered from AGO complexes and meet the miRNA definition criteria (Meyers *et al.*, 2008), many of them were not readily detected by RNA blots due to their relatively low abundance.

The large number of smRNA sequences and clear AGO preference enabled us to examine the expression of known miRNAs and their AGO preference. We found that precursors of some annotated miRNAs produce smRNAs that cover almost every nucleotide of both the forward and reverse strands of the corresponding genomic region. Moreover, these smRNAs have very low overall expression and do not show any AGO1 preference. We propose that these annotated miRNAs may not be *bona fide* miRNAs but rather siRNAs or the so-called young miRNAs (Fahlgren *et al.*, 2007).

We have confirmed and extended a miRNA processing pattern in plants in which two distinct miRNAs appeared to be encoded by the same precursor (Talmor-Neiman *et al.*, 2006; Axtell *et al.*, 2007). Such a phenomenon of second miRNAs, which we called ‘collateral miRNA’, is quite similar to miRNA clusters and miRNA cistrons in mammalian systems. Collateral miRNAs are likely to have appeared early in plant evolution as they are found not only in eudicots and rice, but also in lower plants. The conserved sequence of collateral miRNA encoded by pre-miR319 in several dicots and monocot suggested its functional conservation.

### Phased smRNAs and nat-siRNAs

We have also discovered many nat-siRNAs and phased smRNAs, along with their tissue-specific expression. The large number of phased smRNAs suggested the presence of many ta-siRNA-like loci or uncovered smRNA classes whose functions await further biochemical characterizations. The detection of phased siRNAs related to NAT pairs extended the notion that the latter may be regulated by PTGS via siRNAs. We believe that our data represent a lower limit and additional siRNAs may be discovered from plants subject to biotic or abiotic stresses. The numerous smRNAs with organ-specific origins and AGO-association preferences provided here can also serve as a resource pool for future identification of other functional smRNAs.

### AGO-associated smRNAs and DNA methylation

One unexpected finding of this work is the discovery of a large proportion of 24-nt smRNAs from the AGO1 complexes. In fact, the association of 24-nt smRNAs with the AGO1 complex was previously observed but not investigated further (Qi *et al.*, 2006). These 24-nt smRNAs are not contaminations of AGO4-associated ones, because 91% of AGO1-associated 24-nt smRNAs start with ‘U’, whereas 63% of AGO4-associated 24-nt ones have a 5′-first nucleotide of ‘A’. Although both AGO1- and AGO4-associated 24-nt sequences were mostly derived from heterochromatin, transposons and repeat regions, few overlaps were found among their genomic locations. This result suggests that after cleavage by Dicer-like enzymes, 24-nt smRNAs from these regions were sorted into different AGOs according to their 5′-first nucleotides.

Accumulating evidence indicates that AGO4 plays a crucial role in the RNA-directed DNA methylation (RdDM) pathway involving 24-nt endogenous siRNAs (Chan *et al.*, 2004; Zilberman *et al.*, 2004; Qi *et al.*, 2006). Previous reports on AGO1-associated smRNAs mainly focused on the 21-nt species. Our results showed that a considerable amount of the smRNA species bound to AGO1 were 24-nt and mostly with 5′ U. These 24-nt smRNAs were mainly derived from intergenic regions. Also, these 24-nt smRNAs were unrelated in sequence to the 21-nt species but similar to AGO4-associated 24-nt smRNAs in that they were mostly produced from heterochromatin regions and transposons (Figure S6). As Arabidopsis *ago4* mutants did not exhibit obvious morphological phenotypes (Zilberman *et al.*, 2003), the presence of heterochromatin-produced 24-nt smRNAs in the AGO1 complex suggested that AGO1 may share functional redundancy with AGO4 in regulating RdDM.

RNA polymerase IV (Pol IV)/Pol IVa and Pol V/Pol IVb are two plant-specific RNA polymerases involved in TGS. Pol IV mainly produces 24-nt siRNAs that mediate DNA and histone methylation, whereas Pol V is proposed to induce DNA and histone methylation on specific genomic loci (Pikaard *et al.*, 2008; Wierzbicki *et al.*, 2008). Such silencing processes were mediated by the binding of AGO4/AGO6-associated siRNAs to Pol V transcribed RNAs covering the methylation sites (He *et al.*, 2009; Matzke *et al.*, 2009). We found that over 90% of previously reported potential Pol V-dependent 21-nt siRNAs specifically associate with AGO1, implicating AGO1 with Pol V-related functions. This hypothesis is consistent with the finding that AGO1 controls the expression of a small set of transposons (Lippman *et al.*, 2003). Another possibility is that the AGO1-associated potential Pol V-dependent siRNAs may regulate demethylation rather than methylation (Mosher *et al.*, 2008). Exploration of these potential functions of AGO1 proteins remains an important future challenge.

## Experimental procedures

### DNA construction

pBA-P_AGO4_-FLAG-AGO4 was constructed as follows: FLAG-AGO4 cDNA was obtained using the following pair of primers: 5′-AAGGCGCGCCATGGACTACAAGGATGACGATGACAAGGGCATGGATTCAACAAATGGTAACGG-3′ and 5′-TCTTTAATTAACAGAAGAACATGGAGTTGGC-3′. The PCR-derived fragment was cloned into the backbone of the binary vector pBA002a (Zhang *et al.*, 2005) to generate pBA002a-FLAG-AGO4. Approximately 3.7 kb *AGO4* promoter was amplified using 5′-ACCGGATCCAAATAGCAAAAGCTCATTAGAATAG-3′ and 5′-TCTAGACTCCTGCTCAAAGAAACCAAACAA-3′ primers. The resulting fragment of *AGO4* promoter (−3659 to +1) was cloned into pBA002a-FLAG-AGO4 to generate pBA-P_AGO4_-FLAG-AGO4.

### Plant materials and growth conditions

Seeds of transgenic plants expressing FLAG-AGO1 were obtained from Dr David Baulcombe at University of Cambridge (Baumberger and Baulcombe, 2005). *Arabidopsis thaliana* (Col-0) plants were transformed with the binary vector pBA-P_AGO4_-FLAG-AGO4 by the floral-dip method (Zhang *et al.*, 2006a). Homozygous T_3_ progeny of transgenic lines containing a single insert were used. Untransformed WT (Col-0) and transgenic seedlings were grown on MS plates in a growth chamber under 16-h light/8-h dark at 21°C. Ten-day old seedlings were used. Leaves and roots were harvested from 4-week-old plants grown hydroponically under 12-h light/12-h dark at 21°C in MGRL medium (Fujiwara *et al.*, 1992). Flower samples were collected from 6-week-old plants grown on soil in a growth chamber under 12-h light/12-h dark at 21°C. Samples included floral buds, open flowers and fertilized flowers but not siliques.

### Immunoprecipitation and TSP of AGO/smRNA complexes

One gram of plant tissues (seedlings, flowers, leaves or roots) was ground in liquid nitrogen. Protein/smRNA complexes were extracted in 2 ml buffer containing 20 mm 2-amino-2-(hydroxymethyl)-1,3-propanediol (TRIS)–HCl at pH 7.5, 300 mm NaCl, 5 mm MgCl_2_, 5 mm DTT and EDTA-free protease inhibitor. For one-step IP, after removal of insoluble material by centrifugation twice at 16 000 ***g*** for 10 min at 4°C, extracts were incubated with anti-FLAG M2-agarose beads (Sigma, http://www.sigmaaldrich.com/) for 2 h in IP buffer containing 25 mm TRIS–HCl, pH 7.5, 300 mm NaCl, 4 mm MgCl_2_, 0.2% Triton-100 and 100 μm phenylmethylsulfonyl fluoride (PMSF; Sigma). M2-agarose beads were washed three times with IP buffer. FLAG-AGO1/4 protein–smRNA complexes were eluted by incubation with IP buffer containing 100 μg ml^−1^ 3 × FLAG peptide (Sigma) for 1 h at 4°C. RNAs in the immunoprecipitates were recovered with TRIzol reagent. For two-step purification of AGO/smRNA complexes, cleared extracts (3 ml) were fractioned on Superdex 200 10/300 columns (Akta-FPLC, GE Healthcare, http://www.gehealthcare.com/) equilibrated in 50 mm TRIS–HCl, pH 7.5, 150 mm NaCl. The column was eluted with the same buffer and 80 fractions (3 ml per fraction) were collected. Each fraction was divided into two parts; one aliquot (containing 30 μg total protein) was used for immunoprecipitation followed by western blot using a monoclonal antibody against FLAG (Sigma) to identify fractions containing FLAG-AGO1, whereas the other part was for smRNA extraction. The intensity of AGO protein bands was measured by analyzing the film of protein gel blots using NIH ImageJ software (http://rsb.info.nih.gov/ij/). Recovered RNA was analyzed by RNA blots (Zhang *et al.*, 2006b). Blots were hybridized to ^32^P-radiolabled oligonucleotide probes complementary to the smRNAs. Fractions containing FLAG-AGO1/4 were pooled and further immunoprecipitated with 25 μl anti-FLAG M2-Agarose beads (Sigma) for 2 h in the IP buffer. M2-agarose beads were washed three times with the same buffer and FLAG-AGO1/4 protein-smRNA complexes were eluted as described above.

### Cloning of smRNA libraries

Small non-coding RNA libraries were prepared as described (Hafner *et al.*, 2008). Briefly, total RNA or RNA recovered from the two-step purified AGO–smRNA complexes were spiked with a trace amount of ^32^P radioactively labeled RNA size markers (19-nt, CGUACGCGGGUUUAAACGA; 24-nt, CGUACGCGGAAUAGUUUAAACUGU) before size-fractionation on a denatured polyacrylamide gel. The smRNAs were eluted from excised gel slices with three volumes (v/w) of RNase-free 0.4 m NaCl by incubating the tube overnight at 4°C with constant agitation. Eluted smRNAs were precipitated overnight at −20°C after the addition of three volumes of absolute ethanol. The smRNAs recovered were ligated to chemically pre-adenylated 3′ adapter (AppTCGTATGCCGTCTTCTGCTTG-L) overnight on ice by incubating in the reaction mix [50 μm TRIS–HCl, pH 7.6; 10 μm MgCl_2_; 10 μm 2-mercaptoethanol; 0.1 mg ml^−1^ acetylated BSA (Sigma), 15% (v/v) aqueous DMSO, 2.5 μm adenylated 3′ adapter oligodeoxynucleotide, 0.05 μg μl^−1^ Rnl2(1–249)K227Q]. The ligation products of the smRNA-3′ adapter were further size-fractioned and purified before ligation with 5′ adapter (rGrUrUrCrArGrArGrUrUrCrUrArCrArGrUrCrCrGrArCrGrArUrC) for 1 h at 37°C in a mixture of 25 μm TRIS–HCl, pH 7.6, 5 μm MgCl_2_, 5 μm 2-mercaptoethanol, 0.1 mg ml^−1^ acetylated BSA (Sigma), 15% (v/v) aqueous DMSO, 2.5 μm 5′ adapter oligodeoxynucleotide and 0.05 μg μl^−1^ T4 RNA ligase 1 (Rnl1) (Fermentas, http://www.fermentas.com/). The ligated products of 5′ adapter–smRNA–3′adapter were then used as templates for RT-PCR reactions to generate cDNA libraries. The PCR products within the exponential phase of amplification were used for Illumina deep sequencing. The smRNA sequencing data sets are available at the Gene Expression Omnibus (GSM707678–GSM707691).

### Mapping and annotation of smRNAs

After trimming adaptor sequences, smRNAs with lengths between 19- and 28-nt were selected and mapped to the Arabidopsis genomic sequences (TAIR9 version). Sequences from different samples were normalized by the number of total reads with perfect genomic matches and the normalized clone numbers (reads per million) were used. The genomic features of smRNAs were defined by the same version of genome annotation files. Known miRNA sequences were downloaded from miRBase release 15 and other non-coding RNAs were selected from the annotation file.

### Determination of AGO dominancy for smRNAs

Clone numbers of smRNAs from different samples were first normalized by the total clone numbers of perfectly mapped smRNAs. If the normalized clone number of a smRNA in one AGO sample is five times more than that in the other AGO sample, the smRNA was considered as dominantly associated with that AGO protein.

### Identification of miRNAs

Small non-coding RNAs perfectly mapped to non-transposon intergenic regions were used for miRNA prediction. For each smRNA, the surrounding genomic sequences with extension of 10-nt at either the 5′ or 3′ end of the smRNA and extension of 40-nt to 300-nt with 20-nt increments at the other end were extracted and subjected to secondary structure prediction using mfold software (Zuker, 2003). SmRNAs whose precursor sequence possess good hairpin-shaped secondary structure were selected as miRNA candidates if they had both a clear preference for AGO1 protein and cloned miRNA* sequences, had higher expression than other smRNAs derived from the same precursors, and more than 80% of smRNAs on the precursor were derived from the putative miRNA or miRNA* locus.

### Prediction of phased smRNAs and nat-siRNAs

Small-interfering RNAs with identical lengths and mapping consecutively to the intergenic regions of the Arabidopsis genome were selected. We defined phased smRNAs as those having no fewer than three smRNAs in a series with at least one smRNA had a pairing smRNA mapped to the other strand of the genome with 2-nt overhang at the 3′ end (Figure S9). For cases with missing smRNAs, if the smRNA series had no more than three uncloned smRNAs of the same length between any two cloned smRNAs and a pairing antisense smRNA with 2-nt overhang at the 3′ end, the smRNA series was also considered as phased smRNAs. An expression filter was then applied to select phased smRNAs with at least one smRNA of clone number no less than 10, and at least three smRNAs were cloned from the same tissue.

SmRNAs with a raw clone number of no less than10 were used in nat-siRNA identification. We considered smRNAs mapped to the overlapping region of a *cis*-NAT pair as putative nat-siRNAs.

## SUPPORTING INFORMATION

**Figure S1.** Cloning of AGO-associated small non-coding RNAs (smRNAs) and AGO1-associated smRNAs.

**Figure S2.** Coverage comparison of AGO-associated small noncoding RNAs (smRNAs) identified by two-step purification (TSP) with those detected by immunoprecipitation (IP) or reported by Mi *et al.* (2008).

**Figure S3.** Size distribution and first nucleotide preference of small non-coding RNAs (smRNAs) in unfractionated sample and AGO1/4 complexes from leaves, roots and seedlings. For details see legend of Figure 2.

**Figure S4.** Heat map of known microRNAs (miRNAs) with clone numbers fewer than 1000. For details see legend of Figure 4.

**Figure S5.** Phased small non-coding RNA (smRNA) clusters on *TAS1-4*. Bar charts present non-redundant clone numbers of phased smRNAs. For details see legend of Figure 7.

**Figure S6.** Chromosome distribution of AGO1 and AGO4-associated 24-nucleotide (nt) small non-coding RNAs (smRNAs).

**Figure S7.** DNA-dependent RNA polymerase IV (Pol IV) and Pol V dependence of AGO1 and AGO4-associated small non-coding RNAs (smRNAs).

**Figure S8.** Size distribution of small non-coding RNAs (smRNAs) in DNA-dependent RNA polymerase IV (Pol IV) and Pol V samples.

**Figure S9.** Illustration of algorithm used for prediction of phased small non-coding RNA (smRNA) clusters.

**Table S1.** Relative abundance of AGO1/4 proteins in different organs.

**Table S2.** Summary of smRNA datasets from various samples.

**Table S3.** Specificity of AGO1- and AGO4-preferred 24-nucleotide (nt) small non-coding RNAs (smRNAs).

**Table S4.** Expression profiles of known microRNAs (miRNAs).

**Table S5.** Classification of known microRNA (miRNA) precursors.

**Table S6.** A list of new miRNAs (miRNAs) found in AGO1/AGO4 complexes.

**Table S7.** A list of collateral microRNAs (miRNAs) and their AGO-association in various organs.

**Table S8.** A list of iso-microRNAs (iso-miRNAs).

**Table S9.** A list of new phased small non-coding RNA (smRNA) clusters.

**Table S10.** *cis*-Natural antisense transcript pairs (NAT) pairs and small interfering RNAs (siRNAs).

Please note: As a service to our authors and readers, this journal provides supporting information supplied by the authors. Such materials are peer-reviewed and may be re-organized for online delivery, but are not copy-edited or typeset. Technical support issues arising from supporting information (other than missing files) should be addressed to the authors.
