# Supplementary material for: Deep sequencing of small RNAs specifically associated with Arabidopsis AGO1 and AGO4 uncovers new AGO functions
Source: Plant J. 2011 May 10;67(2):292–304. doi: 10.1111/j.1365-313X.2011.04594.x (PMC3135789; doi:10.1111/j.1365-313X.2011.04594.x)
Supplement: Supplementary file 1 [file tpj0067-0292-SD1.pdf]

Supplementary files

Table S1. Relative abundance of AGO1/4 proteins in different organs.

Table S2. Summary of smRNA datasets from various samples.

Table S3. Specificity of AGO1- and AGO4-preferred 24-nt smRNAs.

Table S4. Expression profiles of known miRNAs.

Table S5. Classification of known miRNA precursors.

Table S6. A list of new miRNAs found in AGO1/AGO4 complexes.

Table S7. A list of collateral miRNAs and their AGO-association in various organs.

Table S8. A list of isomiRNAs.

Table S9. A list of new phased smRNA clusters.

Table S10. *Cis*-NAT pairs and siRNAs.

Figure S1. Cloning of AGO-associated smRNAs and AGO1-associated smRNAs.

(A) The smRNAs recovered from AGO complexes were spiked with ^{32}P-labeled internal 21- and 24-nt smRNA controls and traced during the whole cloning processes. Sample orders listed are internal control; 1, AGO1 IP flower; 2, AGO1 TSP flower; 3, AGO1 TSP seedling; 4, AGO4 TSP flower; 5, AGO4 TSP seedling; 6, unfractionated flower and internal control. (B) Ethium-bromide staining of AGO1-associated smRNAs. AGO1-complexes were immunoprecipitated from 4 g of flower or seedling samples with (+) or without (-) FLAG antibody.

Figure S2. Coverage comparison of AGO-associated smRNAs identified by TSP with those

detected by IP or reported by Mi et.al (2008).

X-axis represents smRNA clone numbers; Y-axis represents proportion of comparative smRNA coverage. Comparison of AGO1-associated smRNAs identified by TSP with those detected by IP in flowers (A) and roots (B), as well as comparison of AGO1 and AGO4-associated smRNAs (C and D) recovered by TSP with those reported by Mi *et.al* (2008) are shown. Only smRNAs with perfectly mapped genomic loci were used.

Figure S3. Size distribution and first nucleotide preference of smRNAs in unfractionated sample and AGO1/4 complexes from leaves, roots and seedlings. For details see legend of Figure 2.

Figure S4. Heat map of known miRNAs with clone numbers fewer than 1000. For details see legend of Figure 4.

Figure S5. Phased smRNA clusters on *TAS1-4*.

Bar charts present non-redundant clone numbers of phased smRNAs. For details see legend of Figure 7.

Figure S6. Chromosome distribution of AGO1 and AGO4-associated 24-nt smRNAs.

X-axis gives chromosome positions on a scale of 10,000-nt. The log2 transformed total clone numbers of smRNAs generated from each 10,000-nt region were shown by red bars (for smRNA loci with total clone number great than 10,000) and blue bars (for smRNA loci with total clone

numbers greater than 1,000 but less than 10,000). 1-F, AGO1-associated 24-nt smRNAs in flowers; 1-L, AGO1-associated 24-nt smRNAs in leaves; 1-R, AGO1-associated 24-nt smRNAs in roots; 1-S, AGO1-associated 24-nt smRNAs in seedlings. 4-F, AGO4-associated 24-nt smRNAs in flowers; 4-L, AGO4-associated 24-nt smRNAs in leaves; 4-R, AGO4-associated 24-nt smRNAs in roots; 4-S, AGO4-associated 24-nt smRNAs in seedlings;

Figure S7. Pol IV and Pol V dependence of AGO1 and AGO4-associated smRNAs.

X-axis gives smRNA dependence of Pol IV and Pol V. Pol IV, smRNAs only dependent on Pol IV; Pol V, smRNAs potentially only dependent on Pol V; Pol IV and Pol V, smRNAs depend on both Pol IV and Pol V. Y-axis presents proportion of AGO preference in each category.

Figure S8. Size distribution of smRNAs in Pol IV and Pol V samples.

(A) Size distribution of smRNAs from WT, pol IV and pol V mutant plants. (B) Size distribution of Pol IV-dependent and potentially Pol V-dependent smRNAs. X-axis gives smRNA length (nt) and Y-axis presents total clone number of smRNAs.

Figure S9. Illustration of algorithm used for prediction of phased smRNA clusters.

smRNA sequences are shown in different colors, and gaps between sequenced smRNAs are represented by open rectangle.

Figure S1

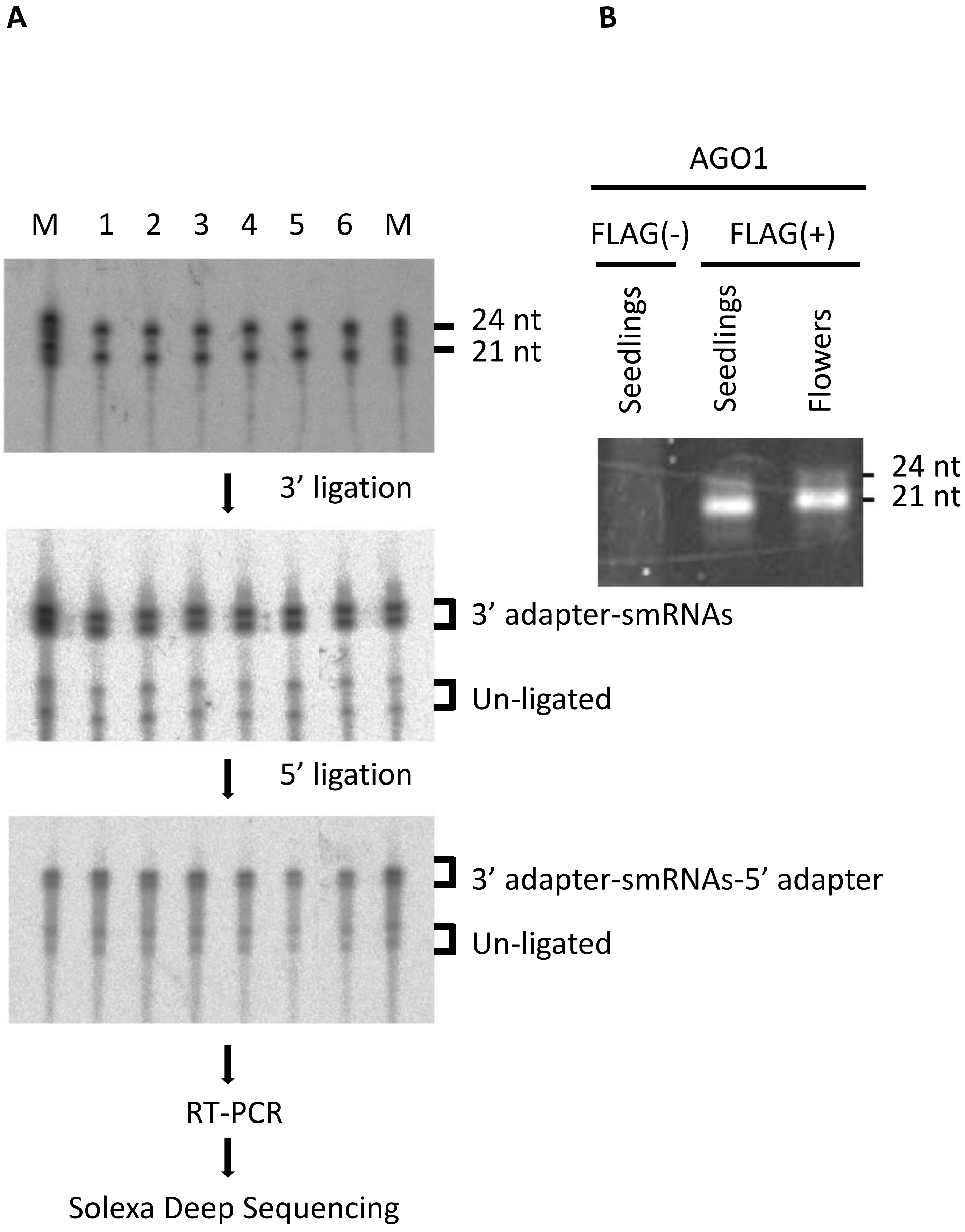
A
M 1 2 3 4 5 6 M
24 nt
21 nt
3’ ligation
3’ adapter-smRNAs
Un-ligated
5’ ligation
3’ adapter-smRNAs-5’ adapter
Un-ligated
RT-PCR
Solexa Deep Sequencing
B
AGO1
FLAG(-)
FLAG(+)
Seedlings
Seedlings
Flowers
24 nt
21 nt

Figure S2

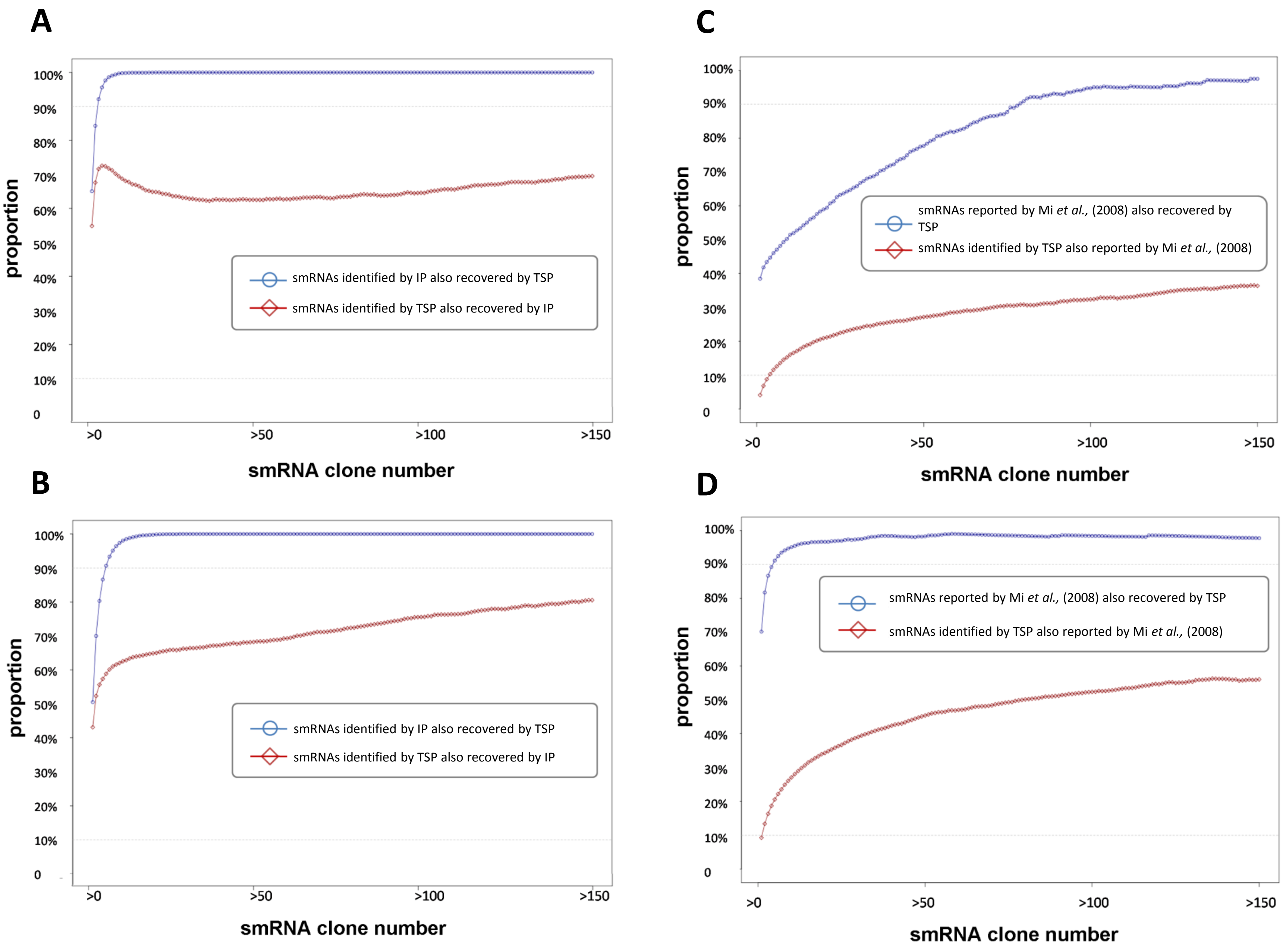
A
proportion
100%
90%
80%
70%
60%
50%
40%
30%
20%
10%
0
>0
>50
>100
>150
smRNA clone number
smRNAs identified by IP also recovered by TSP
smRNAs identified by TSP also recovered by IP
B
proportion
smRNA clone number
smRNAs identified by IP also recovered by TSP
smRNAs identified by TSP also recovered by IP
C
proportion
smRNA clone number
smRNAs reported by Mi *et al.,* (2008) also recovered by TSP
smRNAs identified by TSP also reported by Mi *et al.,* (2008)
D
proportion
smRNA clone number
smRNAs reported by Mi *et al.,* (2008) also recovered by TSP
smRNAs identified by TSP also reported by Mi *et al.,* (2008)

smRNAs in leaves

Figure S3

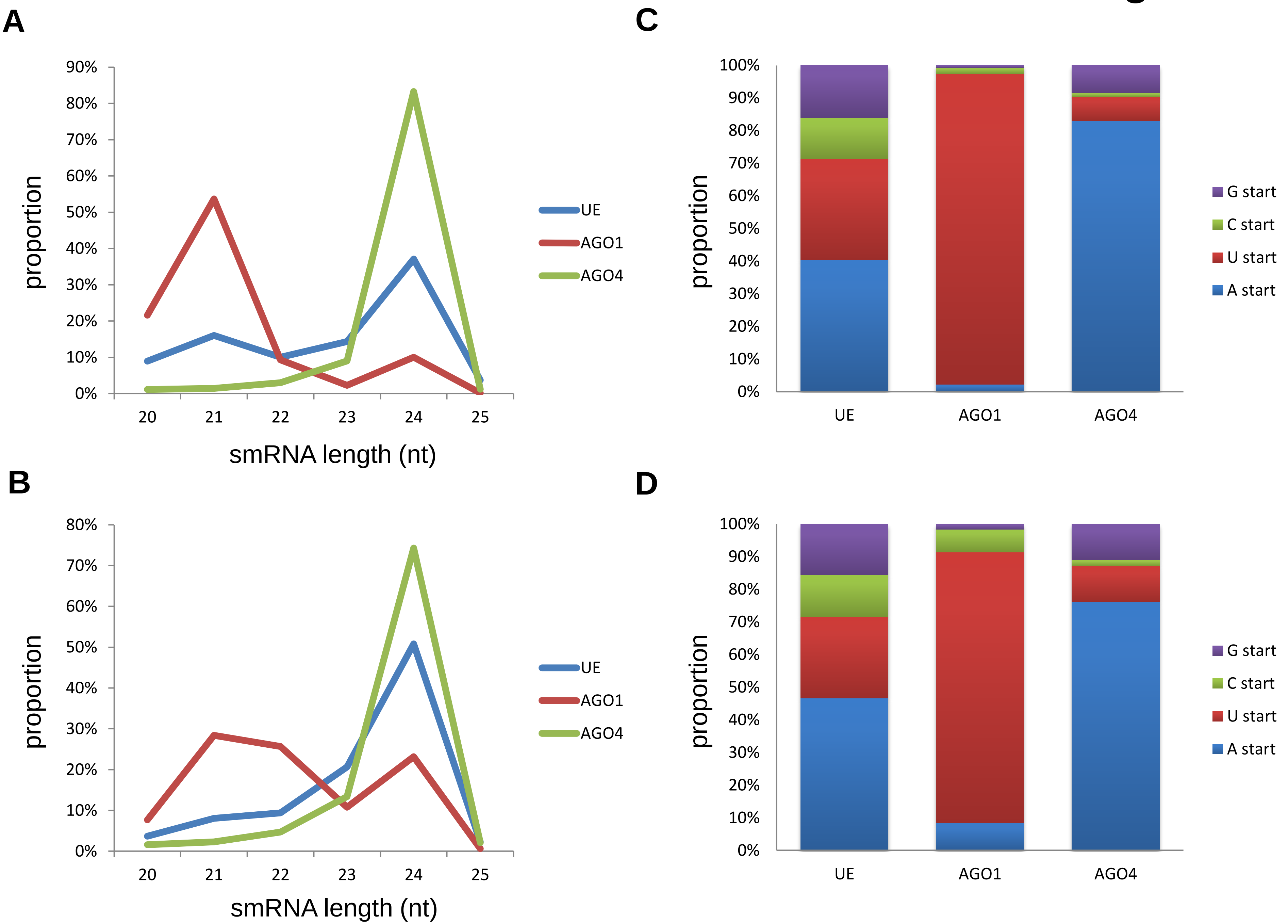

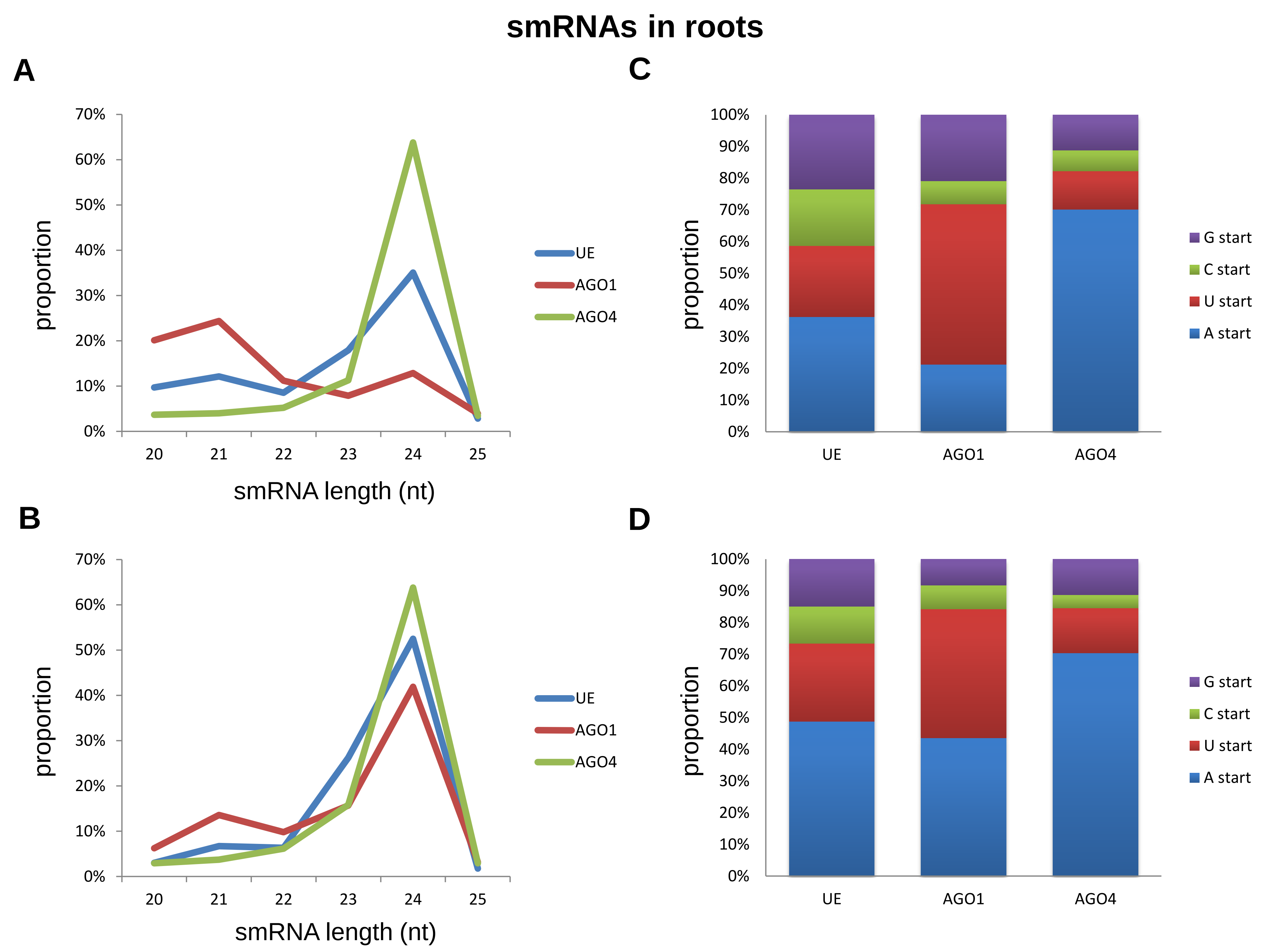
smRNAs in roots
A
B
C
D
proportion
smRNA length (nt)
70%
60%
50%
40%
30%
20%
10%
0%
20
21
22
23
24
25
UE
AGO1
AGO4
100%
90%
80%
70%
60%
50%
40%
30%
20%
10%
0%
G start
C start
U start
A start

smRNAs in seedlings

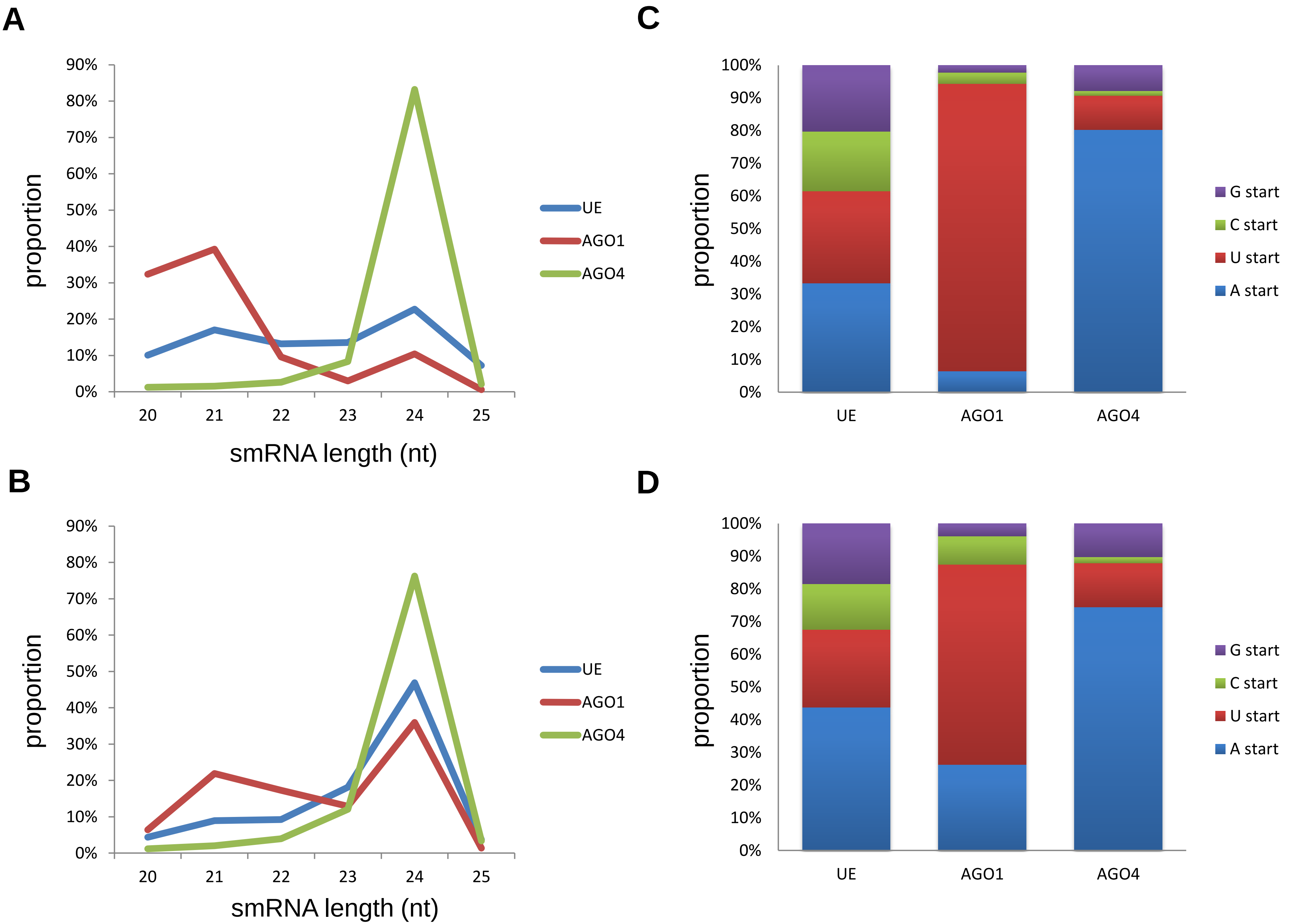

Figure S4

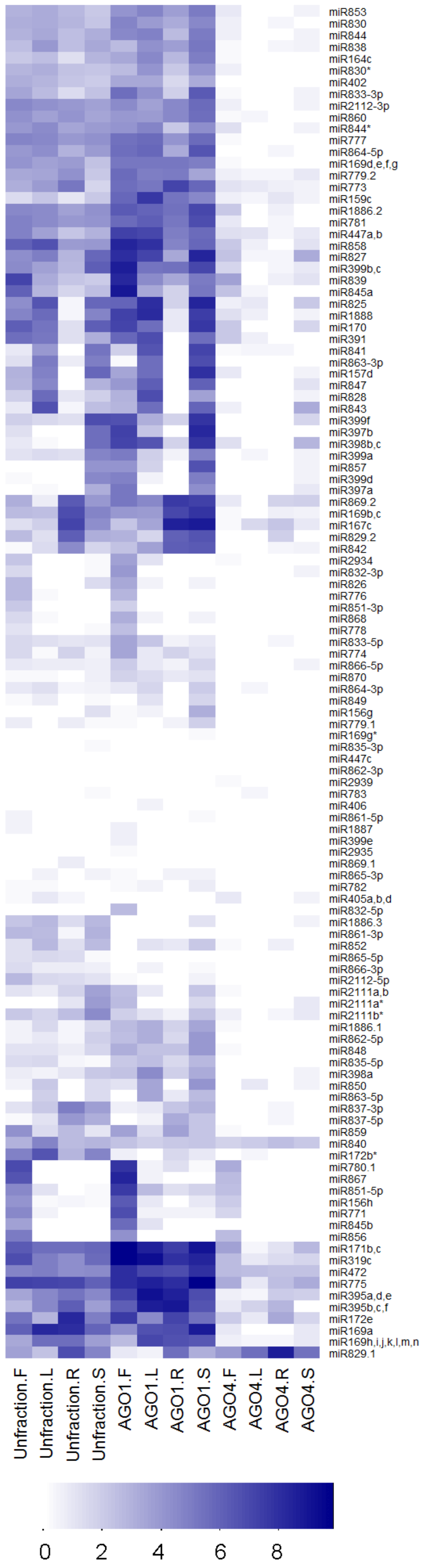
miR853
miR830
miR844
miR838
miR164c
miR830*
miR402
miR833-3p
miR2112-3p
miR860
miR844*
miR777
miR864-5p
miR169d,e,f,g
miR779.2
miR773
miR159c
miR1886.2
miR781
miR447a,b
miR858
miR827
miR399b,c
miR839
miR845a
miR825
miR1888
miR170
miR391
miR841
miR863-3p
miR157d
miR847
miR828
miR843
miR399f
miR397b
miR398b,c
miR399a
miR857
miR399d
miR397a
miR869.2
miR169b,c
miR167c
miR829.2
miR842
miR2934
miR832-3p
miR826
miR776
miR851-3p
miR868
miR778
miR833-5p
miR774
miR866-5p
miR870
miR864-3p
miR849
miR156g
miR779.1
miR169g*
miR835-3p
miR447c
miR862-3p
miR2939
miR783
miR406
miR861-5p
miR1887
miR399e
miR2935
miR869.1
miR865-3p
miR782
miR405a,b,d
miR832-5p
miR1886.3
miR861-3p
miR852
miR865-5p
miR866-3p
miR2112-5p
miR2111a,b
miR2111a*
miR2111b*
miR1886.1
miR862-5p
miR848
miR835-5p
miR398a
miR850
miR863-5p
miR837-3p
miR837-5p
miR859
miR840
miR172b*
miR780.1
miR867
miR851-5p
miR156h
miR771
miR845b
miR856
miR171b,c
miR319c
miR472
miR775
miR395a,d,e
miR395b,c,f
miR172e
miR169a
miR169h,i,j,k,l,m,n
miR829.1
Unfraction.F
Unfraction.L
Unfraction.R
Unfraction.S
AGO1.F
AGO1.L
AGO1.R
AGO1.S
AGO4.F
AGO4.L
AGO4.R
AGO4.S
0
2
4
6
8

Figure S5

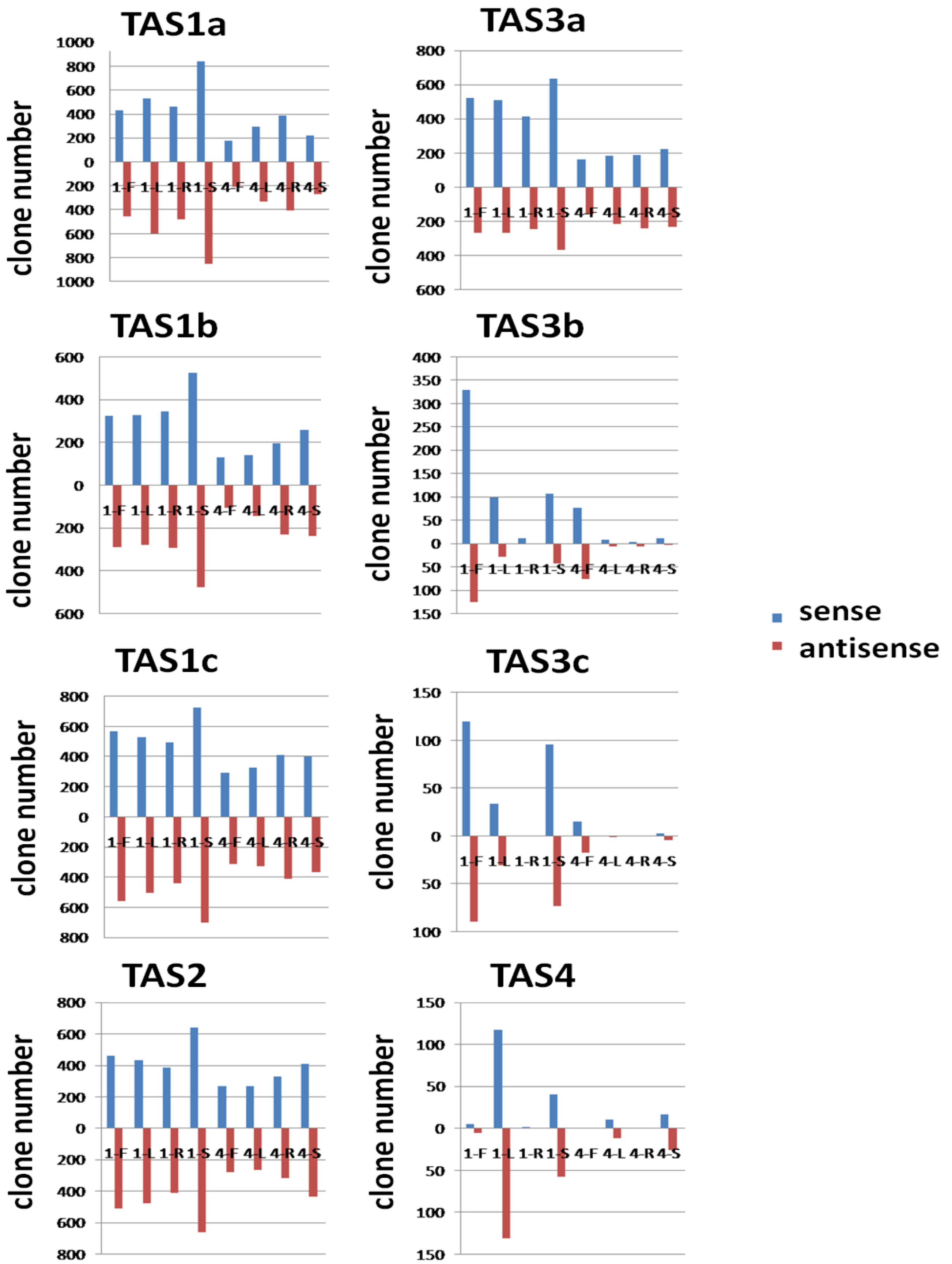
TAS1a
TAS3a
TAS1b
TAS3b
TAS1c
TAS3c
TAS2
TAS4
clone number
1-F 1-L 1-R 1-S 4-F 4-L 4-R 4-S
sense
antisense

Figure S6

chromosome 1

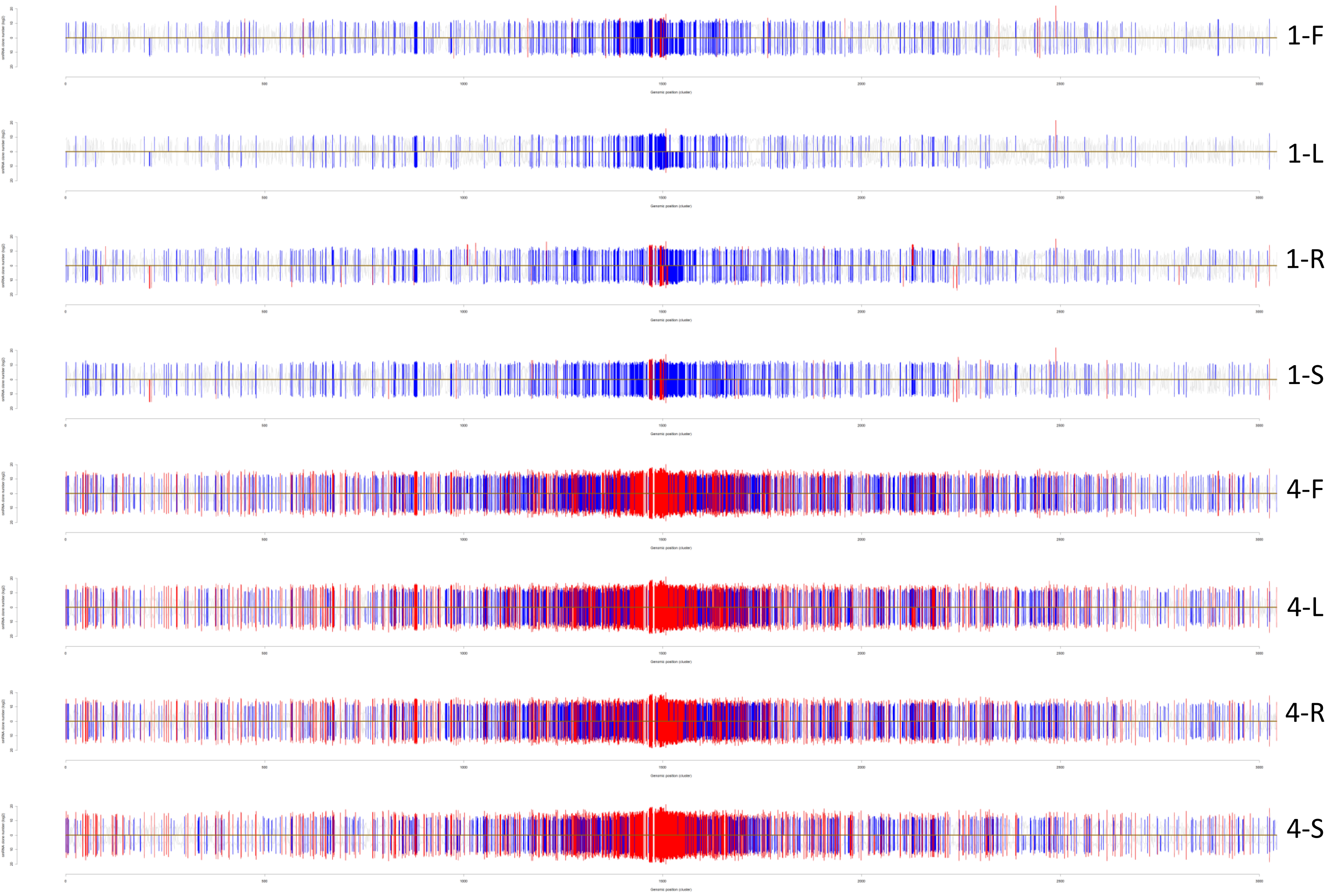

chromosome 2

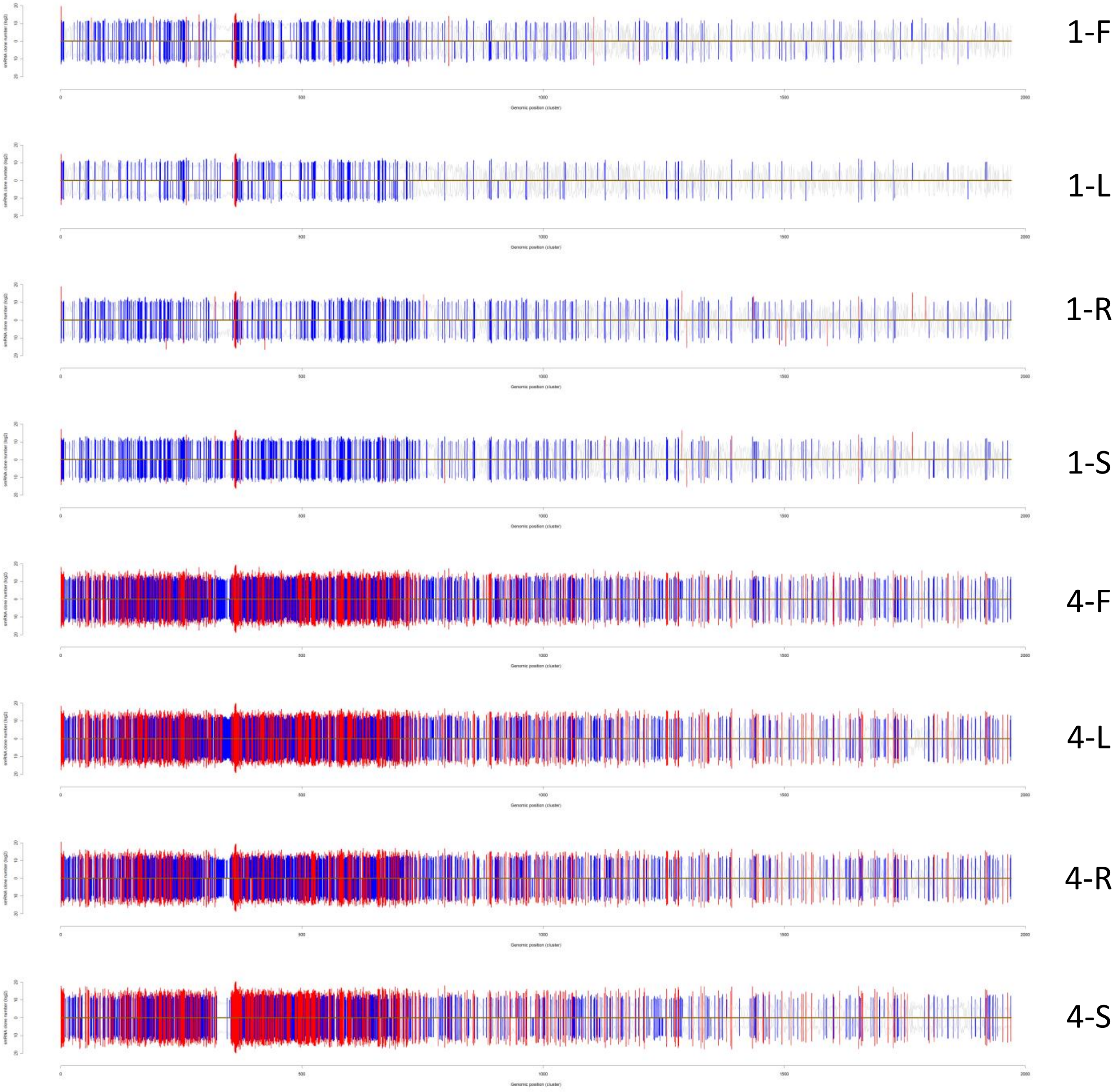

chromosome 3

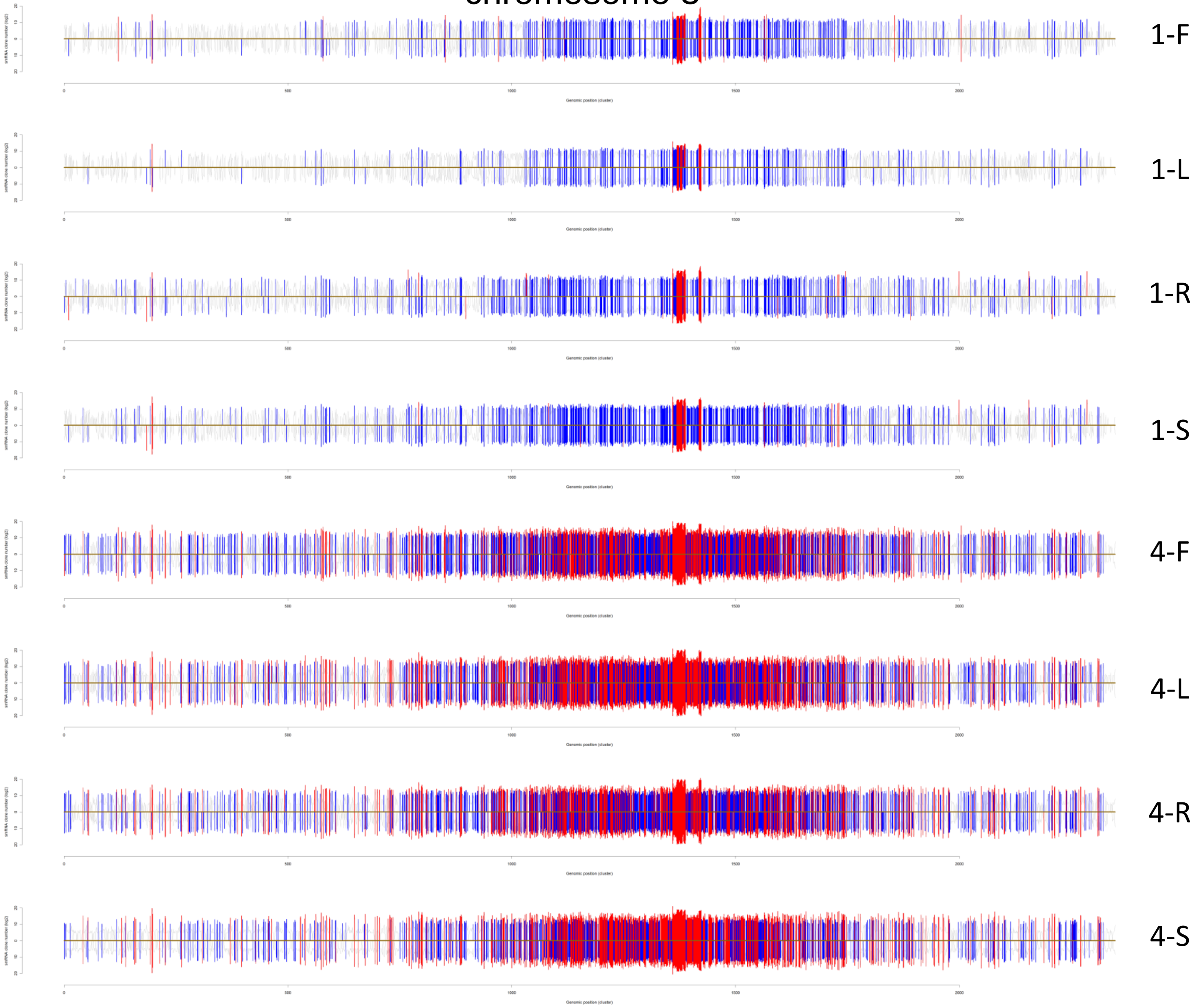

chromosome 4

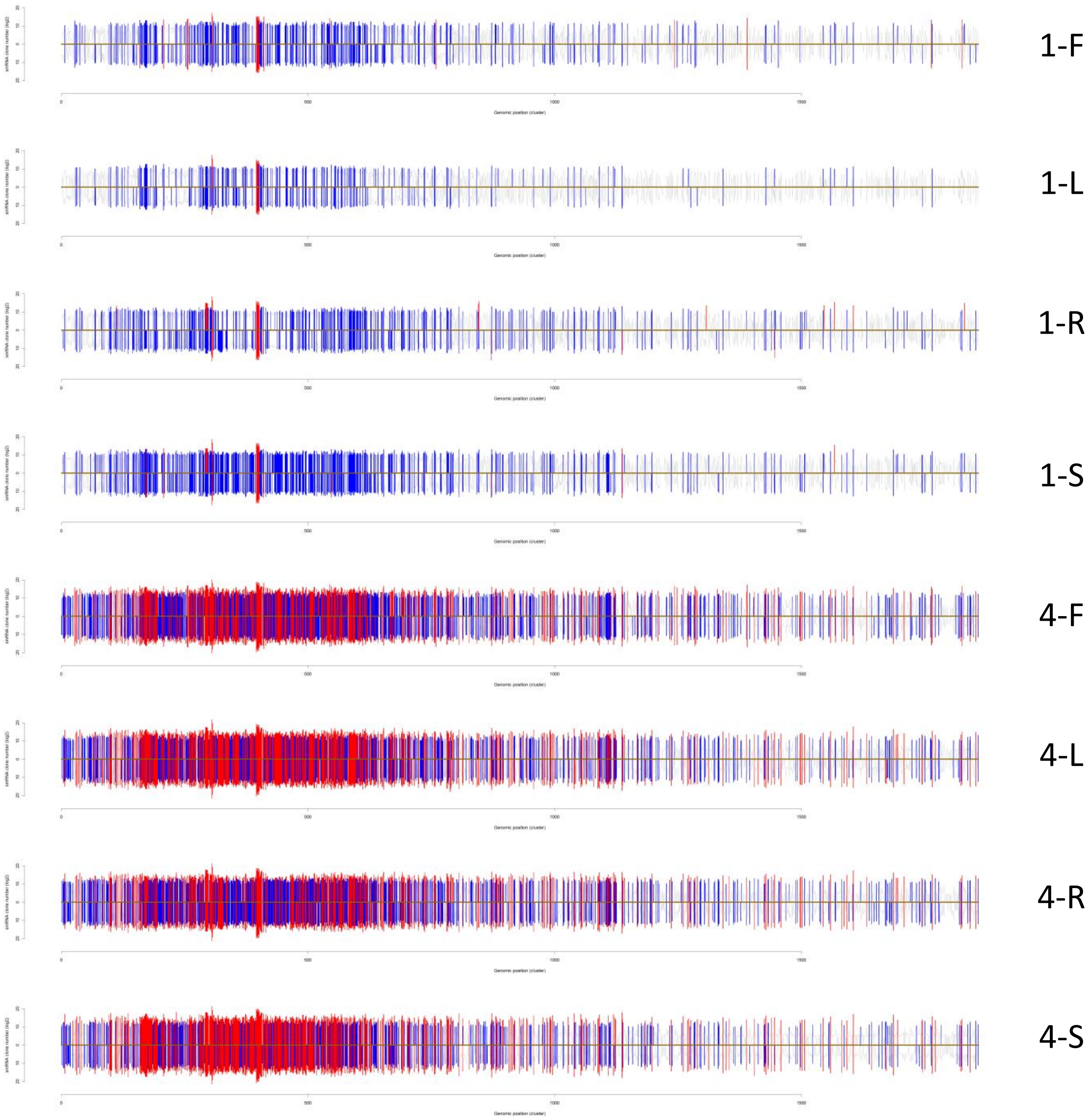

chromosome 5

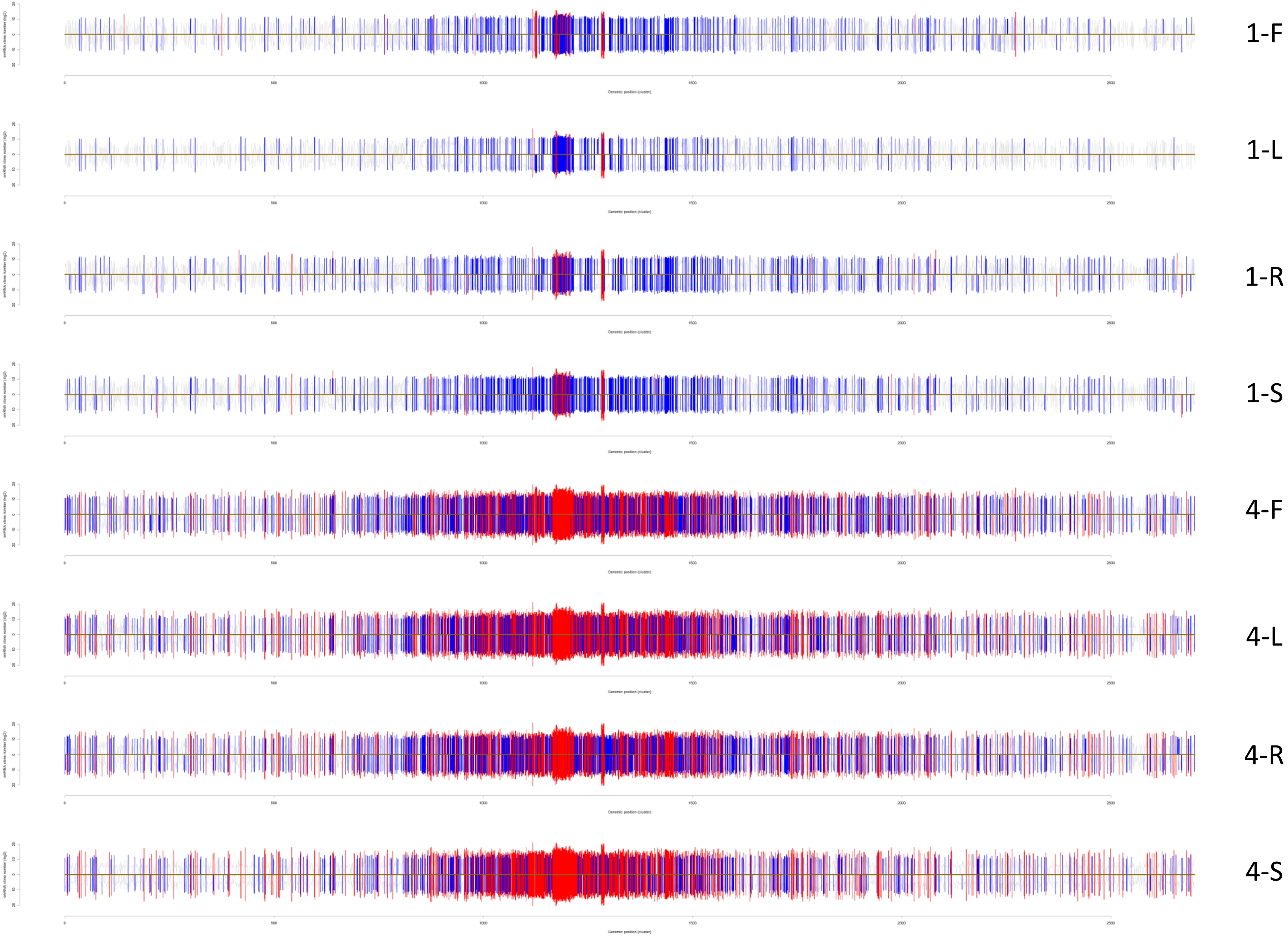

Figure S7

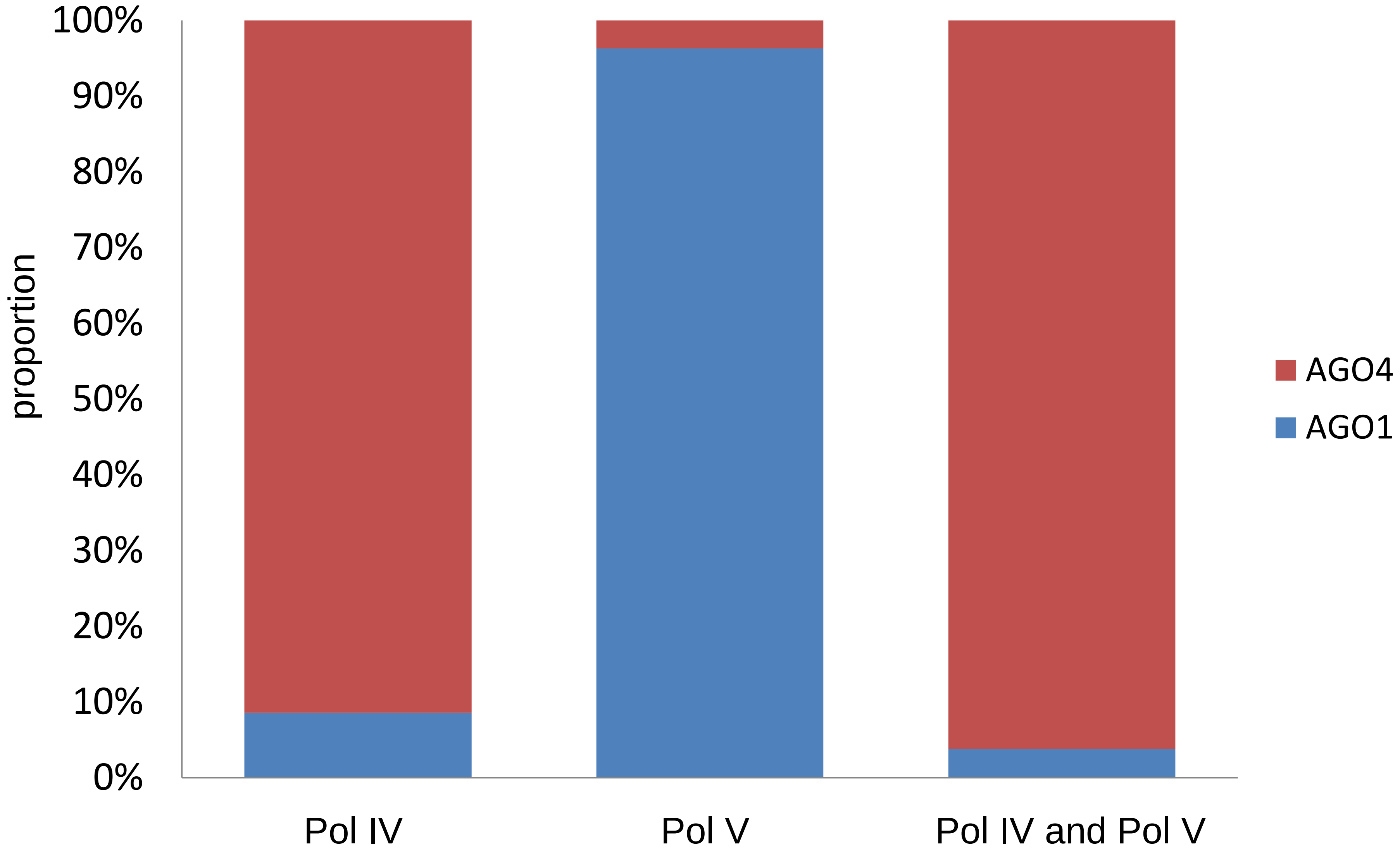
100%
90%
80%
70%
60%
50%
40%
30%
20%
10%
0%
proportion
Pol IV
Pol V
Pol IV and Pol V
AGO4
AGO1

Figure S8

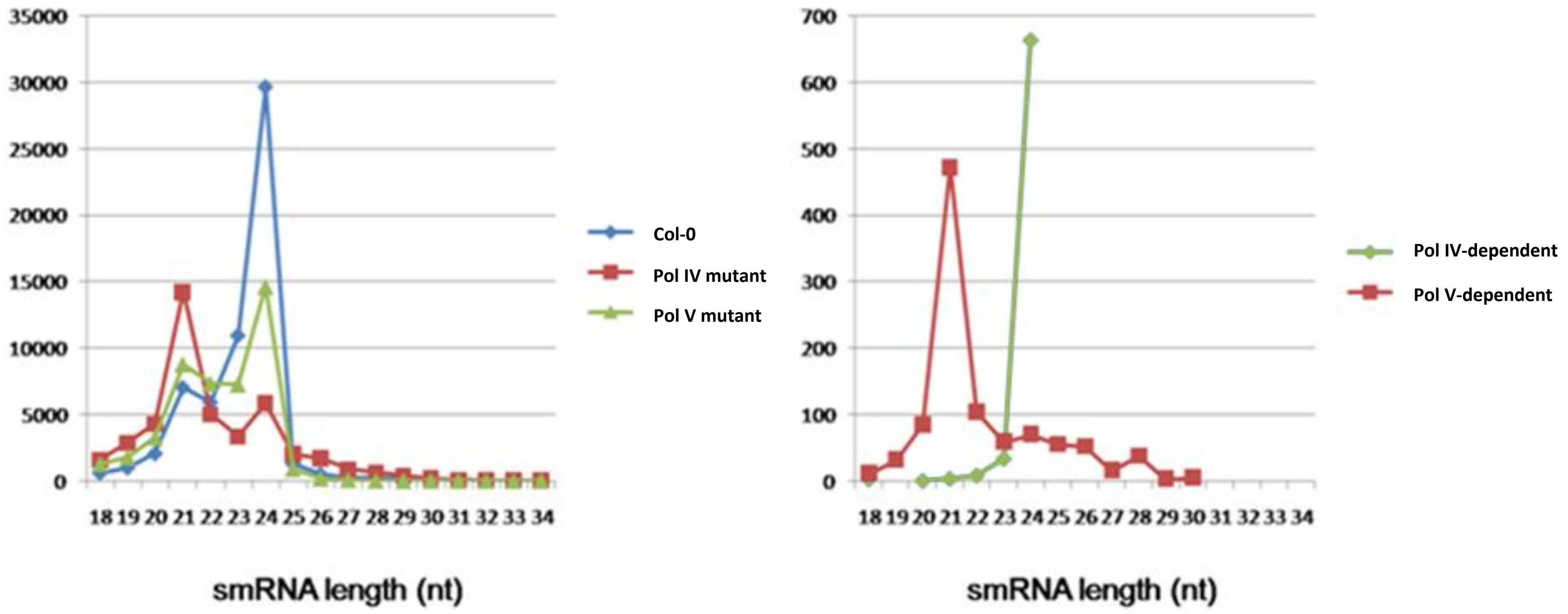
35000
30000
25000
20000
15000
10000
5000
0
18 19 20 21 22 23 24 25 26 27 28 29 30 31 32 33 34
smRNA length (nt)
Col-0
Pol IV mutant
Pol V mutant
700
600
500
400
300
200
100
0
18 19 20 21 22 23 24 25 26 27 28 29 30 31 32 33 34
smRNA length (nt)
Pol IV-dependent
Pol V-dependent

Figure S9

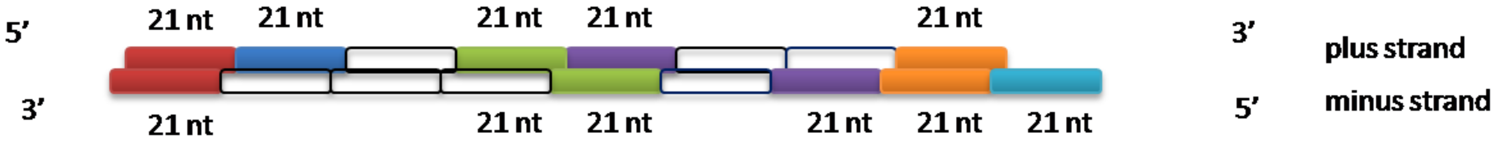
5'
21 nt
21 nt
21 nt
21 nt
21 nt
3'
plus strand
3'
21 nt
21 nt
21 nt
21 nt
21 nt
21 nt
5'
minus strand
