# Supplementary material for: Deep sequencing of small RNAs specifically associated with Arabidopsis AGO1 and AGO4 uncovers new AGO functions
Source: Plant J. 2011 May 10;67(2):292–304. doi: 10.1111/j.1365-313X.2011.04594.x (PMC3135789; doi:10.1111/j.1365-313X.2011.04594.x)
Supplement: Supplementary file 2 [file tpj0067-0292-SD2.doc]

**Supplementary files**

**Figure S1.** Cloning of AGO-associated smRNAs and AGO1-associated smRNAs.

(A) The smRNAs recovered from AGO complexes were spiked with ^32^P-labeled internal 21- and 24-nt smRNA controls and traced during the whole cloning processes. Sample orders listed are internal control; 1, AGO1 IP flower; 2, AGO1 TSP flower; 3, AGO1 TSP seedling; 4, AGO4 TSP flower; 5, AGO4 TSP seedling; 6, unfractionated flower and internal control. (B) Ethium-bromide staining of AGO1-associated smRNAs. AGO1-complexes were immunoprecipitated from 4 g of flower or seedling samples with (+) or without (-) FLAG antibody.

**Figure S2.** Coverage comparison of AGO-associated smRNAs identified by TSP with those detected by IP or reported by Mi et.al (2008).

X-axis represents smRNA clone numbers; Y-axis represents proportion of comparative smRNA coverage. Comparison of AGO1-associated smRNAs identified by TSP with those detected by IP in flowers (A) and roots (B), as well as comparison of AGO1 and AGO4-associated smRNAs (C and D) recovered by TSP with those reported by Mi *et.al* (2008) are shown. Only smRNAs with perfectly mapped genomic loci were used.

**Figure S3.** Size distribution and first nucleotide preference of smRNAs in unfractionated sample and AGO1/4 complexes from leaves, roots and seedlings. For details see legend of Figure 2.

**Figure S4.** Heat map of known miRNAs with clone numbers fewer than 1000. For details see legend of Figure 4.

**Figure S5.** Phased smRNA clusters on *TAS1-4*.

Bar charts present non-redundant clone numbers of phased smRNAs. For details see legend of Figure 7.

**Figure S6.** Chromosome distribution of AGO1 and AGO4-associated 24-nt smRNAs.

X-axis gives chromosome positions on a scale of 10,000-nt. The log2 transformed total clone numbers of smRNAs generated from each 10,000-nt region were shown by red bars (for smRNA loci with total clone number great than 10,000) and blue bars (for smRNA loci with total clone numbers greater than 1,000 but less than 10,000). 1-F, AGO1-associated 24-nt smRNAs in flowers; 1-L, AGO1-associated 24-nt smRNAs in leaves; 1-R, AGO1-associated 24-nt smRNAs in roots; 1-S, AGO1-associated 24-nt smRNAs in seedlings. 4-F, AGO4-associated 24-nt smRNAs in flowers; 4-L, AGO4-associated 24-nt smRNAs in leaves; 4-R, AGO4-associated 24-nt smRNAs in roots; 4-S, AGO4-associated 24-nt smRNAs in seedlings;

**Figure S7.** Pol IV and Pol V dependence of AGO1 and AGO4-associated smRNAs.

X-axis gives smRNA dependence of Pol IV and Pol V. Pol IV, smRNAs only dependent on Pol IV; Pol V, smRNAs potentially only dependent on Pol V; Pol IV and Pol V, smRNAs depend on both Pol IV and Pol V. Y-axis presents proportion of AGO preference in each category.

**Figure S8.** Size distribution of smRNAs in Pol IV and Pol V samples.

(A) Size distribution of smRNAs from WT, pol IV and pol V mutant plants. (B) Size distribution of Pol IV-dependent and potentially Pol V-dependent smRNAs. X-axis gives smRNA length (nt) and Y-axis presents total clone number of smRNAs.

**Figure S9.** Illustration of algorithm used for prediction of phased smRNA clusters.

smRNA sequences are shown in different colors, and gaps between sequenced smRNAs are represented by open rectangle.

**Table S1. Relative abundance of AGO1/4 proteins in different organs.**

**Table S2. Summary of smRNA datasets from various samples.**

**Table S3. Specificity of AGO1- and AGO4-preferred 24-nt smRNAs.**

**Table S4. Expression profiles of known miRNAs.**

**Table S5. Classification of known miRNA precursors.**

**Table S6. A list of new miRNAs found in AGO1/AGO4 complexes.**

**Table S7. A list of collateral miRNAs and their AGO-association in various organs.**

**Table S8. A list of isomiRNAs.**

**Table S9. A list of new phased smRNA clusters.**

**Table S10. *Cis-*NAT pairs and siRNAs.**
